# Activation of the human chemokine receptor CX3CR1 regulated by cholesterol

**DOI:** 10.1126/sciadv.abn8048

**Published:** 2022-06-29

**Authors:** Minmin Lu, Wenli Zhao, Shuo Han, Xiaowen Lin, Tingyu Xu, Qiuxiang Tan, Mu Wang, Cuiying Yi, Xiaojing Chu, Weibo Yang, Ya Zhu, Beili Wu, Qiang Zhao

**Affiliations:** 1 State Key Laboratory of Drug Research, Shanghai Institute of Materia Medica, Chinese Academy of Sciences, 555 Zuchongzhi Road, Pudong, Shanghai 201203, China.; 2 CAS Key Laboratory of Receptor Research, Shanghai Institute of Materia Medica, Chinese Academy of Sciences, Shanghai 201203, China.; 3 University of Chinese Academy of Sciences, No. 19A Yuquan Road, Beijing 100049, China.; 4 School of Pharmaceutical Science and Technology, Hangzhou Institute for Advanced Study, UCAS, Hangzhou, China.; 5 School of Life Science and Technology, ShanghaiTech University, Shanghai 201210, China.; 6 Lingang Laboratory, Shanghai 200031, China.; 7 Zhongshan Institute for Drug Discovery, SIMM, CAS, Zhongshan, China.

## Abstract

As the only member of the CX3C chemokine receptor subfamily, CX3CR1 binds to its sole endogenous ligand CX3CL1, which shows notable potential as a therapeutic target in atherosclerosis, cancer, and neuropathy. However, the drug development of CX3CR1 is hampered partially by the lack of structural information. Here, we present two cryo–electron microscopy structures of CX3CR1-G_i1_ complexes in ligand-free and CX3CL1-bound states at 2.8- and 3.4-Å resolution, respectively. Together with functional data, the structures reveal the key factors that govern the recognition of CX3CL1 by both CX3CR1 and US28. A much smaller conformational change of helix VI upon activation than previously solved class A GPCR-G_i_ complex structures is observed in CX3CR1, which may correlate with three cholesterol molecules that play essential roles in conformation stabilization and signaling transduction. Thus, our data deepen the understanding of cholesterol modulation in GPCR (G protein–coupled receptor) signaling and provide insights into the diversity of G protein coupling.

## Teaser

The CX3CL1-bound and ligand-free CX3CR1-G_i1_ structures reveal cholesterol modulation mechanisms in receptor activation.

## INTRODUCTION

Chemokines are a family of small protein ligands that play critical roles in the recruitment and activation of immune cells by binding to the cognate chemokine receptors (CKRs). According to the number and distribution of conserved cysteines at the N terminus, chemokines are divided into four subfamilies: CC, CXC, CX3C, and XC chemokines; their corresponding receptors are named CCRs, CXCRs, CX3CRs, and XCRs, respectively (*1*). The recognition of chemokines and CKRs presents a promiscuous mechanism: Many CKRs could bind to multiple chemokines and one chemokine can interact with different receptors (*2*). However, the only member of the CX3CR subfamily, CX3CR1, recognizes its sole endogenous ligand CX3CL1 to play essential roles in the cross-talk between monocytes and endothelial cells in the periphery as well as microglia and neurons in the central nervous system (*3*–*5*). CX3CR1 primarily couples to G_i_ upon activation, subsequently mediating multiple signaling pathways, causing cell migration, angiogenesis, and apoptosis resistance (*6*, *7*). CX3CR1 participates in numerous physiological processes and is involved in many important human diseases, such as atherosclerosis, rheumatoid arthritis, neurodegenerative diseases, and cancer, making it an attractive therapeutic target (*8*–*11*). In addition, US28, a virus-encoded G protein–coupled receptor (GPCR) with 29% sequence identity with CX3CR1, could also bind to CX3CL1 and serve in viral infection (*12*). As the promiscuity property of chemokines and CKRs is an obstacle in drug development, CX3CR1 presents the unique advantage as a potential drug target. However, the progress of drug development targeting CX3CR1 is relatively slow and challenging (*13*–*15*). To date, only one high-affinity small-molecule inhibitor, AZD8797, and an anti-CX3CR1 nanobody are in phase 1 clinical trials to treat cancer pain and kidney disease, respectively (*16*, *17*).

Several chemokine-bound CKR-G_i_ complex structures in CC and CXC subfamilies have been solved recently, including crystal structures as well as cryo–electron microscopy (cryo-EM) structures of US28-CX3CL1 and US28-CX3CL1.35 (*18*–*25*). However, the chemokine recognition specificity mechanism, especially in the CX3C subfamily, remains unclear, and the lack of CX3CR1 structural information hinders the understanding of the activation mechanism of human CX3C CKR (*23*, *24*). To reveal the molecular details of ligand recognition and receptor activation for CX3CR1, we solved two cryo-EM structures of CX3CR1-G_i1_ complexes in the ligand-free and CX3CL1-bound states, respectively. Together with mutagenesis data, the structural information demonstrates the specific ligand binding mode in the CX3C chemokine subfamily and the essential roles of cholesterol molecules in receptor activation, providing structural and functional evidence for ligand recognition and cholesterol modulation in CX3CR1 signaling.

## RESULTS

### Overall structures of CX3CR1-G_i1_ and CX3CR1-CX3CL1-G_i1_ complexes

**Fig. 1.**
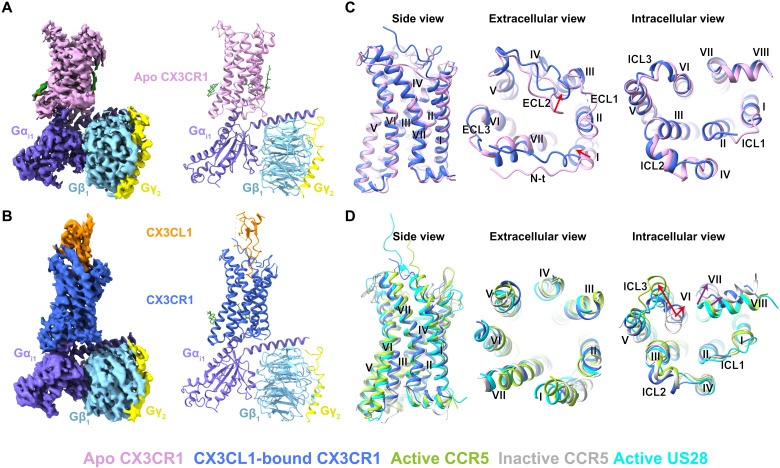
Overall structures of CX3CR1-G_i1_ and CX3CR1-CX3CL1-G_i1_ complexes. (**A** and **B**) Cryo-EM maps and structures of CX3CR1-G_i1_ (A) and CX3CR1-CX3CL1-G_i1_ (B), colored according to chains. The receptor CX3CR1 in two states is colored pink and blue, respectively. The chemokine CX3CL1 is colored dark orange. The three subunits in G_i1_ are colored slate, sky blue, and yellow, respectively. Cholesterol is colored green. (**C**) Superposition of CX3CR1 in two complex structures. The red arrows indicate the movements of the N terminus (N-t), helix I, and ECL2 of CX3CR1 upon CX3CL1 binding. (**D**) Structural comparison of CX3CL1-bound CX3CR1 with CCR5 and US28. The structures of inactive CCR5 [Protein Data Bank (PDB) ID: 4MBS], active CCR5 (PDB ID: 7F1Q), and active US28 (PDB ID: 7RKM) are colored gray, yellow green, and cyan, respectively. The red arrows indicate the outward movements of the intracellular ends of helix VI of active CCR5 and CX3CR1 with A^6.33^ as reference. The purple arrows indicate conformational changes of helix VII and helix VIII of CX3CR1 compared with active CCR5.

To increase the protein yield and homogeneity of the complex, three mutations (I120^3.43^L, C221^ICL3^S, and M250^6.54^V) [superscript indicates residue numbering using the Ballesteros-Weinstein nomenclature (*26*)] were introduced into the wild-type (WT) CX3CR1, and 40 residues (C316 to L355) were truncated from the C terminus of the receptor (fig. S1, A and H). Among these mutations, I120^3.43^L and M250^6.54^V were introduced by rationally screening to improve protein yield and thermostability (fig. S1, C and D). C221^ICL3^S was introduced to prevent the formation of potential mispairing disulfide bonds during expression and purification. These modifications showed little effect on receptor activation as the engineered construct exhibited a similar calculated CX3CL1 potency (the negative logarithm of the median effective concentration, pEC_50_) value compared to the WT (fig. S1E). To facilitate the formation of the CX3CR1-CX3CL1 complex, the CX3CL1 C terminus and the CX3CR1 N terminus were connected by a 28-residue (14 × Gly-Ser) linker. In addition, a pair of disulfide cross-linking mutations between CX3CR1 and CX3CL1 (L176^ECL2^C in CX3CR1 and G35C in CX3CL1) was introduced to further stabilize the complex (fig. S1, B, G and H). The fused form of CX3CR1-CX3CL1 with a cross-linking disulfide bond showed constitutive activity with similar maximal efficacy (*E*_max_) in comparison with WT receptor, which is also observed in the CX3CR1-CX3CL1 fusion protein without cross-linking (fig. S1F). These data suggested that neither fusion nor cross-linking has an obvious effect on the efficacy of CX3CL1 and its corresponding receptor. The human dominant negative Gα_i1_ (DNGα_i1_) and WT human Gβ_1_/γ_2_ were used to form a stable G_i1_ heterotrimer, and it appears that the CX3CR1 could couple to G_i_ with or without the presence of agonist CX3CL1. Using single-particle cryo-EM, the ligand-free and CX3CL1-bound CX3CR1-G_i1_ complex structures were determined with an overall resolution of 2.8 and 3.4 Å, respectively (Fig. 1, A and B; fig. S2 and S3; and table S1).

Although the structures of CX3CR1 in two complexes exhibit a similar conformation with the receptor Cα (residues T31 to Y305) root mean square deviation of 1.4 Å, the superposition of CX3CR1 in two states reveals obvious differences in the extracellular part of the receptor. Compared to the ligand-free complex, the N terminus of the receptor moves closer to the center axis of the helical bundle and the extracellular loop 2 (ECL2) is pushed away upon CX3CL1 binding (Fig. 1C). In contrast to the extracellular side, the intracellular part of two CX3CR1 structures adopts a similar conformation with the helices well overlaid (Fig. 1C). In the previously solved GPCR–G protein complex structures, the large outward movement of helix VI was observed, which is considered as a hallmark of active conformation in class A GPCRs. However, the G_i1_-coupled CX3CR1 structures in both ligand-free and chemokine-bound states exhibit a much smaller outward movement of helix VI in the intracellular side (Fig. 1D). Compared with the CCR5 structure in an inactive state [Protein Data Bank (PDB) ID: 4MBS], the intracellular end of helix VI in the active CX3CR1 structure only shows a 2.3-Å outward movement (A^6.33^ as reference), which is much smaller than that in the active CCR5 structure (8.2 Å) (PDB ID: 7F1Q) and G_i_-bound US28 structure (6.7 Å) (PDB ID: 7RKM). This limited displacement results in a narrower space between the intracellular end of helix III and helix VI in CX3CR1, and thus, to accommodate Gα_i_, helix VII and helix VIII of CX3CR1 shift away from the helical center, with a 5.8-Å outward movement of helix VII (Y^7.53^ as reference) and a 3.7-Å shift of helix VIII (K^8.49^ as reference) compared with the active CCR5 structure (Fig. 1D). The distinct conformation of CX3CR1 results in a unique coupling mode of G_i_. The calculated coupling interface between G_i1_ and CX3CR1 is about 900 Å^2^, which is larger than that in CCR5-G_i_ (826 Å^2^) and US28-G_i_ (790 Å^2^) complex structures. Both the particular conformation of CX3CR1 and the G protein coupling mode suggest the different activation mechanisms of CX3CR1.

### CX3CL1 recognition by CX3CR1

**Fig. 2.**
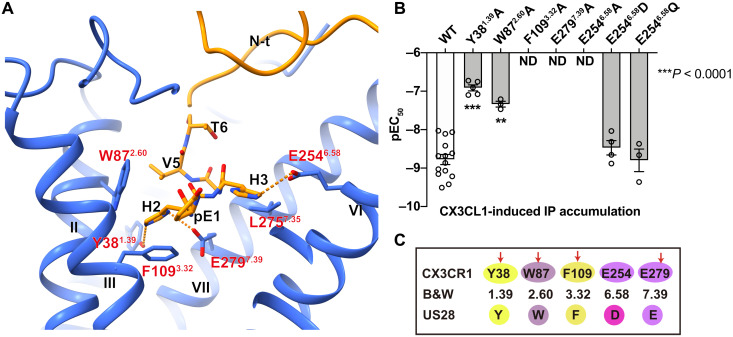
Binding mode of the N terminus of CX3CL1 (pE1 to T6). (**A**) Binding pocket of the N terminus of CX3CL1 in CX3CR1. The residues pE1 to T6 of CX3CL1 are shown as sticks. The polar interactions are shown as orange dashed lines. (**B**) CX3CL1-induced IP accumulation of the CX3CR1 mutants using a chimeric Gα protein Gα_qi5_. Bars are represented with calculated CX3CL1 potency (pEC_50_) of CX3CR1 WT and mutants. Data are shown as means ± SEM (bars) from at least three independent experiments (*n*) performed in triplicate with individual data points shown (dots). ND, not determined. **P < 0.001 and ***P < 0.0001 by one-way analysis of variance (ANOVA) followed by Dunnett’s post-test, compared with the response of WT. See table S2 for detailed statistical evaluation and expression levels. (**C**) Sequence alignment of key residues involved in CX3CL1 binding in CX3CR1 and US28, respectively.

CX3CL1 binds to CX3CR1 in a two-site model: The globular part of CX3CL1 sits on the extracellular region of CX3CR1 and occupies chemokine recognition site 1 (CRS1), while the N terminus of CX3CL1 [pE1(pyroglutamate 1)-T6] forms a hook-like conformation and inserts into the helical bundle, CRS2 (Figs. 1B and 2A) (*27*). The interactions in CRS1 are ambiguous because of the weak density, whereas CRS2 is clearly modeled, allowing the analysis of the binding mode of CX3CL1 with the CX3CR1 transmembrane domain (fig. S3C). In CRS2, the N-terminal hook (pE1-H2-H3-G4-V5-T6) of CX3CL1 reaches deep into the transmembrane helical core and forms extensive polar interactions with CX3CR1 (Fig. 2A). The interface is verified by an inositol phosphate (IP) accumulation assay using a chimeric Gα protein Gα_qi5_, which replaces the last five C-terminal residues of Gα_q_ with those in Gα_i_ and converts G_i_-related signaling into a G_q_ readout (Fig. 2B, fig. S4A, and table S2) (*28*). Among these residues in the receptor binding pocket, two acidic residues appear to be very important for recognition of its chemokine. E254^6.58^ forms a salt bridge with H3 of CX3CL1, and our data suggested that breaking the salt bridge by the E254^6.58^A mutation drastically impairs the CX3CL1 binding and CX3CR1 activation (Fig. 2B, fig. S4A, and table S2) (*29*). Moreover, E254^6.58^Q and E254^6.58^D can rescue the signaling to a comparable level to WT, which may be due to the hydrophilic environment provided by glutamine or aspartic acid (Fig. 2B, fig. S4A, and table S2). Another conserved glutamic acid, E279^7.39^, that was reported to play important roles in chemokine binding also forms extensive polar interactions with H2 and the main chain of G4-V5 of CX3CL1 (*30*, *31*). Replacing E279^7.39^ with alanine completely abolishes the signaling of CX3CR1 (Fig. 2B, fig. S4A, and table S2). Besides, H2 is further stabilized by the polar interaction with Y38^1.39^, and the mutant Y38^1.39^A results in a 62-fold reduction of the median effective concentration (EC_50_) of CX3CL1 (Fig. 2B, fig. S4A, and table S2). In addition to the above polar interactions, two other residues, F109^3.32^ and W87^2.60^, form hydrophobic contacts with H2 and V5 of CX3CL1, respectively. The IP accumulation results suggest that F109^3.32^A significantly impairs CX3CL1-induced CX3CR1 activation and W87^2.60^A displays a 17-fold reduction of EC_50_ of CX3CL1 (Fig. 2B, fig. S4A, and table S2). Sequence alignment results suggest that the above key residues involved in CRS2 are conserved across CX3CR1 and US28, including Y^1.39^, W^2.60^, F^3.32^, and E^7.39^, which can account for the high affinity of CX3CL1 with these two receptors (Fig. 2C) (*23*).

**Fig. 3.**
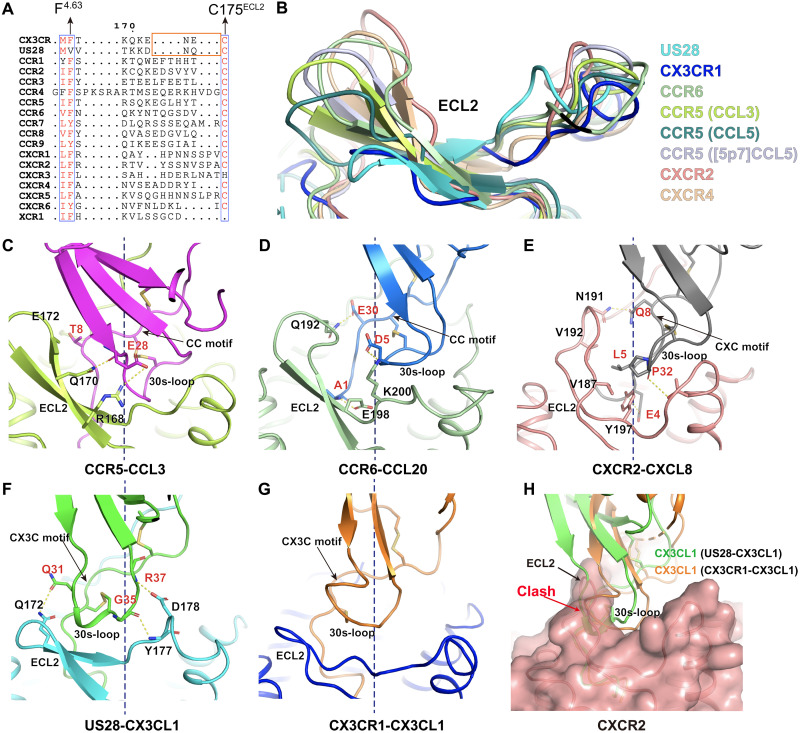
Interactions between ECL2 and chemokines in different subfamilies. (**A**) Sequence alignment of human CKRs and US28. The orange rectangle indicates the tip region of ECL2 β-hairpin. (**B**) Superposition of ECL2s in different CKR structures of US28-CX3CL1 (PDB ID: 4XT1), CCR6-CCL20-G_io_ (PDB ID: 6WWZ), CCR5-CCL3-G_i1_ (PDB ID: 7F1Q), CCR5-CCL5-G_i1_ (PDB ID: 7F1R), CCR5-5P7-RANTES (PDB ID: 5UIW), CXCR2-CXCL8-G_i1_ (PDB ID: 6LFO), and CXCR4-vMIPII (PDB ID: 4RWS). (**C** to **G**) Interactions between CKR-ECL2 and chemokines in different subfamilies. The polar interactions are shown as yellow dashed lines. The dashed black line represents the central axis of helical bundle. (C) Interactions between ECL2 of CCR5 and CCL3. (D) Interactions formed by ECL2 of CCR6 and CCL20. (E) Interactions between ECL2 of CXCR2 and CXCL8. (F) Interactions formed by ECL2 of US28 and CX3CL1. (G) Structure of ECL2 of CX3CR1 and CX3CL1. (**H**) Superposition of the CX3CR1-CX3CL1-G_i1_, US28-CX3CL1 (PDB ID: 4XT1), and CXCR2-CXCL8-G_i1_ (PDB ID: 6LFO) structures, showing the steric clash between the ECL2 of CXCR2 and the 30s-loop of CX3CL1 in both US28-CX3CL1 and CX3CR1-CX3CL1-G_i1_ structures. CXCR2 is presented as surface, and CX3CL1 is shown as a cartoon. The black arrow indicates the ECL2 of CXCR2, and the red arrow indicates the clash region.

Although the overall binding mode of chemokines follows a common “two-site” model, the detailed interactions vary between the 30s-loop of chemokine and the ECL2 of receptors in different subfamilies, which may explain the unique recognition mechanism between CX3CL1 and CX3CR1. Combined with sequence alignment, the structure superposition of solved chemokine-CKR complexes displayed a noticeable difference in the length of ECL2 β-hairpins and binding modes with the chemokine 30s-loop region. The ECL2s of CC and CXC CKRs are longer than those of CX3CR1 and US28 (Fig. 3, A and B). Meanwhile, the extended ECL2 of CCR5, CCR6, CXCR2, and CXCR4 forms multiple interactions with related 30s-loop regions of chemokines (Fig. 3, C to F). In contrast, the β-hairpin of CX3CR1 ECL2 is shorter and forms little interactions with the 30s-loop of CX3CL1 (Fig. 3G). In the solved chemokine-CKR structures, the related location of the second cysteine in the cysteine motif of chemokine is conserved by the disulfide bonding interaction with the chemokine core domain (Fig. 3, C to G). Caused by the distinct cysteine motif, the 30s-loop presents a different orientation due to another disulfide bonding interaction with the first cysteine in the cysteine motif of chemokine (Fig. 3, C to G). In both CX3CR1-CX3CL1 and US28-CX3CL1 complex structures, the 30s-loop shifts toward ECL2 with the CX3C cysteine motif (Fig. 3, F and G). Coincidentally, the shorter ECL2 of CX3CR1 and US28 suits the conformation of the 30s-loop of CX3CL1, which is distinct from other subfamilies (Fig. 3, B and F to H). The comparison of the binding modes of ECL2 and the 30s-loop shows that the CX3C motif fixes the 30s-loop of CX3CL1 closer to the ECL2 and would form a steric clash with the longer tip of the β-hairpins in the other CKRs (Fig. 3H). Thus, the differences in ECL2 between different subtypes of CKRs might serve as one of the key determinants for chemokine selectivity.

### Cholesterols modulate CX3CR1 activation

**Fig. 4.**
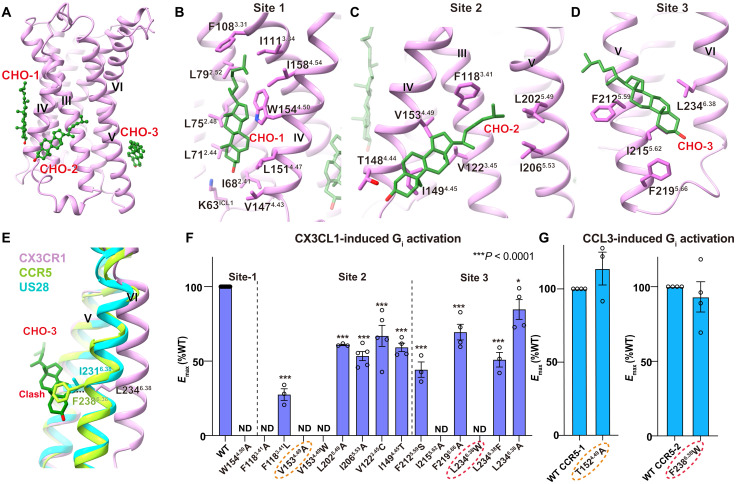
Cholesterol-binding sites in CX3CR1. (**A**) Binding sites of three cholesterol (CHO) molecules in CX3CR1. Cholesterols are shown as green ball-sticks, and CX3CR1 is shown in cartoon representation. (**B** to **D**) Binding pockets of cholesterol 1 (B), cholesterol 2 (C), and cholesterol 3 (D), respectively. Cholesterols and residues in the pockets are shown as sticks. (**E**) Comparison of helix VI of CX3CR1 with the active CCR5 and G_i_-bound US28. (**F**) CX3CL1-induced G protein activation of the CX3CR1 WT and mutants in cholesterol-binding pockets tested via the TRUPATH biosensor, using Gα_i1_-Rluc8, Gβ_3_, and Gγ_9_-GFP2. Data are shown as means ± SEM (bars) from at least three independent experiments (*n*) performed in triplicate with individual data points shown (dots). *P < 0.01 and ***P < 0.0001 by one-way ANOVA followed by Dunnett’s post-test, compared with the response of WT. See table S3 for detailed statistical evaluation and expression levels. The mutants tested in both CX3CR1 and CCR5 are labeled with dashed ovals. (**G**) CCL3-induced G protein activation of the CCR5 WT and mutants tested via the TRUPATH biosensor, using Gα_i1_-Rluc8, Gβ_3_, and Gγ_9_-GFP2. Data are means ± SEM (bars) from at least three independent experiments (*n*) performed in triplicate with individual data points shown (dots). See table S4 for detailed statistical evaluation and expression levels.

Cholesterol is reported as an essential component in the cell membrane and plays a critical role in regulating receptor function by binding to specific sites in GPCRs (*32*–*34*). A site-specific cholesterol consensus motif has been identified in β_2_-adrenergic receptor (β_2_AR) and serves to stabilize the receptor conformation in many GPCRs (*35*). However, the functional relationship between cholesterol and CKRs remains unknown. In the CX3CR1-G_i1_ complex structures, three cholesterol molecules (cholesterol 1 to 3) are observed in the specific binding pockets (site 1 to 3) of helices II to VI (Fig. 4, A to E). Different from cholesterol 1, which corresponds to the previously observed one in β_2_AR, cholesterol 2 and cholesterol 3 are found for the first time in G_i_-coupled GPCR complex structures.

To confirm the roles of these cholesterols in CX3CR1 activation, we carried out the TRUPATH assay of WT and mutants in sites 1 to 3 (*36*). Cholesterol 1 binds to the proximal intracellular part of helices II, III and IV, and forms interactions with surrounding hydrophobic residues, including L75^2.48^, L79^2.52^, F108^3.31^, I111^3.34^, L151^4.47^, and W154^4.50^ (Fig. 4B). Among these residues, the highly conserved W154^4.50^ in class A GPCRs plays the most important role in cholesterol binding by CH-π stacking interaction, which accounts for the universal binding of cholesterols at this site (Fig. 4B). The alanine substitution of W154^4.50^ results in the inability of CX3CR1 coupling with G_i_, supporting the significance of cholesterol 1 in mediating the CX3CR1 signaling (Fig. 4F, fig. S4B, and table S3).

Cholesterol 2 inserts into site 2 composed of helices III to V by extensive hydrophobic interactions. The iso-octyl tail is embedded in the cleft surrounded by F118^3.41^, L202^5.49^, P203^5.50^, and I206^5.53^ (Fig. 4C). The TRUPATH results suggest that F118^3.41^ plays a central role in CX3CL1-induced receptor activation and cholesterol binding. The substitution of F118^3.41^ with alanine or a less bulky residue such as leucine abolishes G_i_ coupling or results in an 80% reduction of the WT *E*_max_, respectively (Fig. 4F, fig. S4C, and table S3). Similar to F118^3.41^, V153^4.49^ has a vital role in the interaction with the tetracyclic sterol ring of cholesterol, because V153^4.49^A mutant abolishes the CX3CL1-mediated CX3CR1 activation (Fig. 4, C and F; fig. S4C; and table S3). To assess the unique contribution of V153^4.49^ to CX3CR1 activation, we generate the substitution of V153^4.49^W from the corresponding residue in US28, which possibly causes the steric hindrance by the bulky side chain, leading to the loss of function of CX3CR1 (Fig. 4F, fig. S4C, and table S3). In contrast, a similar T152^4.49^A mutant of CCR5 displays little effect on G_i_ signaling (Fig. 4G, fig. S4D, and table S4), suggesting the unique role of cholesterol 2 in activating CX3CR1 rather than CCR5.

Similarly, mutants in site 3 also have severe effects on receptor activation. Ring A/B of cholesterol 3 is wedged between I215^5.62^ and L234^6.38^, which restricts the outward shift of helix VI (Fig. 4, D and E). I215^5.62^A significantly reduces the *E*_max_ of CX3CR1, indicating its important role in cholesterol 3 binding and CX3CR1 activation (Fig. 4F, fig. S4F, and table S3). Although L234^6.38^A has little effect on the signaling of CX3CR1 compared to WT, the substitution of L234^6.38^ with phenylalanine, which is located at the corresponding site in CCR5, markedly decreases the *E*_max_ of CX3CR1 induced by CX3CL1 (Fig. 4, E and F; fig. S4G; and table S3). Besides, L234^6.38^W completely abrogates receptor activation, which is due to the steric hindrance with cholesterol (Fig. 4F, fig. S4G, and table S3). In contrast, a similar mutant, F238^6.38^W, has no substantial effect on G_i_ signaling of CCR5 (Fig. 4G, fig. S4H, and table S4). In addition, cholesterol 3 is further stabilized by two phenylalanines, F212^5.59^ and F219^5.66^, and disruption of these interactions leads to the *E*_max_ reduction of CX3CR1 (Fig. 4, D and F; fig. S4F; and table S3). The different behaviors between CX3CR1 and CCR5 mutants in site 2 and site 3 further confirm the specific binding and the unique conformational stabilization role of cholesterols in CX3CR1. All the results above demonstrate the crucial roles of cholesterols in CX3CR1 activation.

Sequence alignment shows that most residues in site 1 are conserved across human CKRs, while the residues in site 2 and site 3 are less conserved (fig. S5A). Analysis of key residues affecting cholesterol binding in CX3CR1 suggests that only 16.7% (XCR1, CCR8, and CX3CR1) of human CKRs contain the restrictive residue cluster of cholesterol binding in site 2 and also 16.7% (CCR7, CCR8, and CX3CR1) in site 3, demonstrating the particularity of CX3CR1 in cholesterol-modulated activation (fig. S5, B and C). Thus, the unique cholesterol-binding pattern might be specific to particular types of CKRs.

### The different coupling modes of G_i_ to CX3CR1

**Fig. 5.**
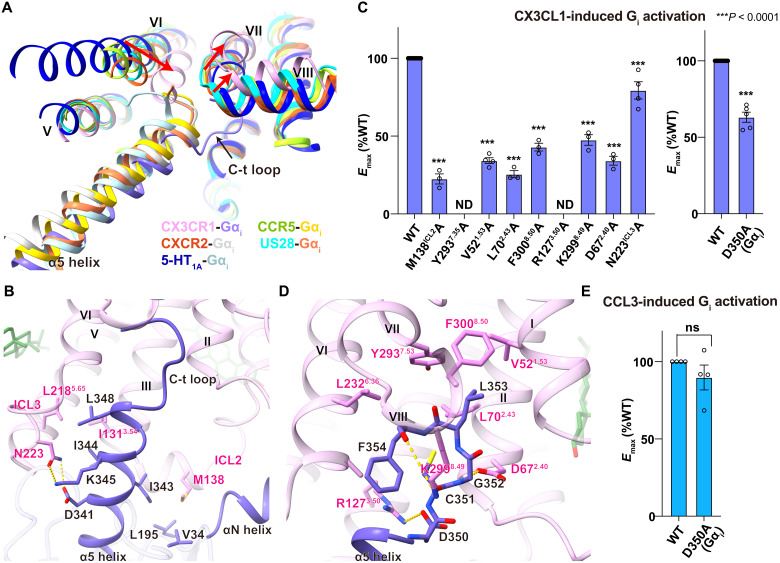
Different coupling modes of G_i_ to CX3CR1. (**A**) Comparison of receptors and G_i_ conformation in CX3CR1 (pink)–G_i1_ (slate), CCR5 (yellow green)–G_i_ (gold), CXCR2 (orange red)–G_i_ (light gray), US28 (cyan)–G_i_ (coral), and 5-HT_1A_ (dark blue)–G_i_ (light blue). The red arrows indicate the conformational changes of helix VI, helix VII, and helix VIII of CX3CR1 compared with other active GPCRs. The black arrow indicates the different coupling pose of the C terminus (C-t) of Gα_i_ in the CX3CR1-G_i1_ complex. (**B**) Interface between α5 helix of Gα_i1_ and CX3CR1, with side chains of Gα_i1_ and CX3CR1 shown in sticks. The polar interactions are shown as yellow dashed lines. (**C**) CX3CL1-induced G protein activation of the WT and mutant CX3CR1 and Gα_i1_ in G protein interface. Data are shown as means ± SEM (bars) from at least three independent experiments (*n*) performed in triplicate with individual data points shown (dots). One-way ANOVA was performed followed by Dunnett’s post-test and compared with WT. The *P* value was defined as ***P < 0.0001. See table S3 for detailed statistical evaluation and expression levels. (**D**) Interface between the C-terminal loop (D350 to F354) of Gα_i1_ and CX3CR1, with side chains of Gα_i1_ and CX3CR1 shown in sticks. The polar interactions are shown as yellow dashed lines. (**E**) CCL3-induced G protein activation of WT CCR5 using WT and mutant Gα_i1_-Rluc8, Gβ_3_, and Gγ_9_-GFP2. Data are means ± SEM (bars) from at least three independent experiments (*n*) performed in triplicate, with individual data points shown (dots). See table S4 for detailed statistical evaluation and expression levels. ns, not significant.

While the binding of cholesterol 3 stabilizes the distinct active conformation of helix VI with limited outward movement, G_i_ adopts a unique interaction mode with CX3CR1. The superposition of the CX3CR1-G_i1_ structure and solved G_i_-coupled CKR structures [CCR5-G_i_ (PDB ID: 7F1S), CXCR2-G_i_ (PDB ID: 6LFM), and US28-G_i_ (PDB ID: 7RKM)] shows a smaller outward displacement of the intracellular end of helix VI (Fig. 5A) (*20*–*22*, *37*). Previously, there are two other receptors (CCR5 and 5-HT_1A_) that show the ability to couple G_i_ without the presence of an agonist. Compared with the cryo-EM structures of apo-CCR5-G_i_ (PDB ID: 7F1S) and apo-5-HT_1A_-G_i_ (PDB ID: 7E2X), helix VI of CX3CR1 in CX3CR1-G_i1_ complex structures exhibits significantly smaller outward movement, presenting a narrower opening of the G protein binding pocket (Fig. 5A) (*20*, *33*). In contrast, helix VI of CCR5 and 5-HT_1A_ show a shift similar to previously solved active GPCR structures, indicating that the unique helix VI movement of CX3CR1 during activation is not likely caused by the lack of an agonist but a receptor-specific conformation in G protein coupling (Fig. 5A). Besides helix VI, the intracellular ends of helix VII and helix VIII shift away from the helix bundle to accommodate G protein, resulting in the C-terminal loop of G protein (D350-C351-G352-L353-F354) adopting a different orientation to helix VII and helix VIII compared with previously solved GPCR-G_i/o_ complex structures (Fig. 5A). The unique conformation of Gα_i_ leads to a more extensive interaction network involving helix I to VIII, the second and third introcellular loops (ICL2 and ICL3) of CX3CR1. Although the specific helical rearrangement to adopt the G_i_ protein, most interactions formed by the α5 helix and CX3CR1 are still conserved in comparison to other class A GPCR-G_i_ complexes (Fig. 5B). For example, L348 and I344 form strong hydrophobic interactions with helix III, helix V, and helix VI (Fig. 5B). The ICL2 of CX3CR1 forms a two-turn helical structure with the hydrophobic side chain of M138^35.54^ penetrating into a pocket involving V34, L194, and I343 of Gα_i_ (Fig. 5B). Furthermore, D341 forms a hydrogen bond with N223^ICL3^ in ICL3 of CX3CR1 (Fig. 5B). The importance of these residues in ICL2 and ICL3 is reflected by the attenuation of CX3CL1-induced *E*_max_ of CX3CR1, as mutants in this region such as M138A and N223A significantly decrease the receptor activation (Fig. 5C; fig. S4, I and J; and table S3).

Despite the similar binding mode of the α5 helix, the C-terminal loop of G_i1_ displays a completely different orientation and forms extensive interactions with CX3CR1 in comparison with other G_i_-coupled structures (Fig. 5D). The negatively charged residue D350 in Gα_i_ forms a salt bridge with R127^3.50^ in the conserved “DRY” motif of helix III (Fig. 5D). The specifically bound cholesterol molecules in CX3CR1 potentially stabilize its unique conformation of helix III and helix VI, leading to an unstretched conformation of R127^3.50^ different from that in known class A G protein–bound active structures (Fig. 5D and fig. S6A). Both the IP accumulation and TRUPATH assay results reveal that the R127^3.50^A mutant abolishes the activity of the receptor (Fig. 5C; fig. S4, A and J; and tables S2 and S3). In addition, the D350A mutant in Gα_i_ blunts its coupling ability with WT CX3CR1 while showing little effect on CCR5 activation (Fig. 5, C and E; fig. S4, K and L; and tables S3 and S4). All the results indicate the essential roles of R127^3.50^ and the salt bridge in the G_i_ signaling of CX3CR1. Except for the particular salt bridge with the DRY motif, there are also several polar interactions that stabilize the CX3CR1-G_i_ complex, including the hydrogen bonding interaction between G352 of Gα_i_ and D67^2.40^ of CX3CR1 and the ionic interaction between the carboxyl group of F354 of Gα_i_ and K299^8.49^ of CX3CR1 (Fig. 5D). This was confirmed according to our TRUPATH results, as D67^2.40^A in CX3CR1 also showed greatly damaged signaling (Fig. 5, C and D; fig. S4J; and table S3). Moreover, there is also a hydrophobic cluster formed by V52^1.53^, L70^2.43^, Y293^7.53^, and F300^8.50^ of CX3CR1 that surrounds L353 in the C-terminal loop of Gα_i_ (Fig. 5D). According to our mutagenesis data, Y293^7.53^A mutant abolishes signaling completely, and the alanine mutation of the other three residues in this pocket (V52^1.53^A, L70^2.43^A, and F300^8.50^A) also shows the significantly impaired activation of CX3CR1 (Fig. 5C, fig. S4I, and table S3).

## DISCUSSION

As the only member of the CX3CR subfamily, the structure of the CX3CL1-bound CX3CR1-G_i1_ complex deepens our understanding of the chemokine recognition mechanism for the human CKR superfamily. The chemokines in different subfamilies show a similar structure architecture with the conserved core domain and the unstructured N terminus. The reported chemokine-CKR complex structures have provided some hints that correlated with chemokine-CKR selectivity, including the differently charged distribution or conserved motifs in the CKR N terminus and chemokine core domain interface (*20*, *22*). In our structures, we found the unique structural feature of the ECL2 region in CX3CR1 as well as another CX3CL1-recognized receptor US28 (*23*). The less-extended ECL2 suits the conformation of the 30s-loop in CX3C chemokine, which shows the distinct binding mode in this region from other chemokine-CKR subfamilies. This may indicate one of the complicated recognition mechanisms of the CX3C chemokine-CRK subfamily. Excluding the common ECL2 feature, the residues related to the CX3CL1 N-terminal hook interaction are almost conserved in CX3CR1 and US28, accounting for the high affinity of CX3CL1 with these two receptors (Fig. 2C).

**Fig. 6.**
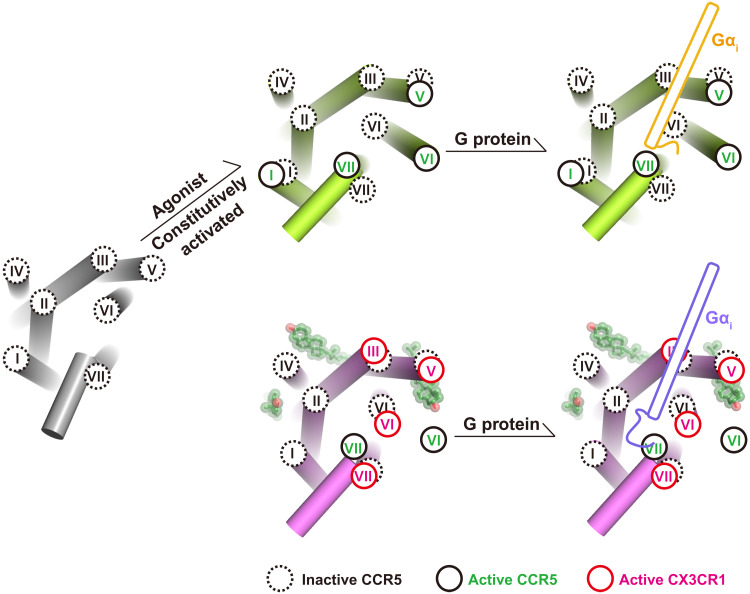
The diverse active conformation of CX3CR1 and CCR5. The helices are represented as cylinders. Cholesterol molecules are displayed in ball-stick representation with transparent spheres. The intracellular ends of transmembrane helices of inactive CCR5 (PDB ID: 4MBS), active CCR5 (PDB ID: 7F1Q), and active CX3CR1 are shown in black dashed circles, black solid circles, and red solid circles, respectively. The C termini of Gα_i_ of CCR5-G_i_ and CX3CR1-G_i_ complexes are colored gold and slate, respectively.

It has been well known that cholesterol is an allosteric modulator of GPCRs. Previously, there is one common cholesterol-binding site identified in many GPCRs; our structures show the distinct coupling mode of G_i_ that is modulated by the cholesterol. The two cholesterol molecules might be the main contributors in stabilizing the unique active conformation of CX3CR1 with a closer distance between helix III and helix VI in the intracellular end (Figs. 1D and 4E). To create a binding cavity for G protein, the intracellular ends of helix VII and helix VIII are pushed away from the helical center (Fig. 5A). In the published G protein coupling structures, a similar smaller opening of helix VI has been firstly observed in the prostaglandin E receptor EP4-G_s_ complex structure (*38*). Unlike the cholesterol modulation in CX3CR1, the nonconserved S^6.48^ in the toggle switch and F^2.39^, which interacts with Y^7.35^ in the N/DPxxY motif, result in the unique binding mode of G_s_ with prostaglandin E receptor EP4 (*38*). Together with the uncanonical conformation of Gα_i_ in CX3CR1-G_i1_ complex structures, these divergent coupling modes of G protein engagement with individual receptors suggest the complicated activation mechanism for class A GPCRs (Fig. 6).

In addition to the different G protein coupling modes, the active CX3CR1 also presents various conformational changes of key conserved activation motifs in class A GPCRs, such as D^3.49^R^3.50^Y^3.51^, C^6.47^W^6.48^xP^6.50^, N^7.49^P^7.50^xxY^7.53^, and P^5.50^I^3.40^F^6.44^ (*39*). When comparing these motifs in active CX3CR1 with those in both active and inactive CCR5, R^3.50^ in the DRY motif stretches to helix VI and forms a hydrogen bond interaction with Y^5.58^ in active-state CCR5 (fig. S6). However, it keeps a downward conformation and forms a salt bridge with D350 of Gα_i1_ in CX3CR1-G_i_ complexes (fig. S6, A and B). Moreover, the highly conserved residue W^6.48^ acting as a “toggle switch” and the “PIF/Y” motif display rotamer conformational changes during CCR5 activation, while the motifs of CX3CR1 more likely behave like the inactive-state CCR5 (fig. S6, C and D). These structural features also indicate a different activation mechanism of CX3CR1.

In summary, the CX3CL1-bound CX3CR1 structure reveals molecular details for the recognition mechanism of CX3CL1. Combined with extensive mutagenesis studies, the distinct cholesterol-binding sites deepen our knowledge about the modulation of cholesterols in GPCRs. In addition, the related conformational change between CX3CR1 and G_i_ provides insight into the distinct coupling modes of G proteins with individual GPCRs.

## MATERIALS AND METHODS

### Cloning and protein expression

Modified human CX3CR1 (residues 1 to 315) with three mutations (I120^3.43^L, C221^ICL3^S, and M250^6.54^V) and a C-terminal Twin-Strep-tag was cloned into the pFactBac1 vector (Invitrogen), which was used to form the complex with G_i1_ in the ligand-free state and defined as construct-1. To facilitate the complex formation of CX3CL1 with CX3CR1, the chemokine CX3CL1 (residues 1 to 76) was fused to the N terminus of CX3CR1 with a 28-residue linker (14 × Gly-Ser), and an additional disulfide bond between CX3CR1 (L176^ECL2^C) and CX3CL1 (G35C) was added. Modified CX3CL1-CX3CR1 followed by the Twin-Strep-Tag at the C terminus was defined as construct-2. A DNGα_i1_ with S47C, G202T, G203A, E245A, and A326S (*40*, *41*) and 6 × His-tagged human Gβ_1_/Gγ_2_ were cloned into pFastBac1 and pFastBac Dual vector (Invitrogen), respectively. The codon-optimized DNA sequences and all primer sequences used in this study are included in table S5. Construct-1/conctruct-2, DNGα_i1_, and 6 × His-tagged human Gβ_1_/Gγ_2_ were coexpressed in HighFive insect cells (Invitrogen, catalog no. B85502), and the virus was prepared using the Bac-to-Bac system (Invitrogen). The cells at a density of 1.5 × 10^6^ cells ml^−1^ were infected with high-titer viral stocks (>10^9^ viral particles/ml) at a multiplicity of infection ratio of 1:2:2 for CX3CR1/CX3CL1-CX3CR1, DNGα_i1_, and Gβ_1_/Gγ_2_. Cells were grown at 27°C for 48 hours and then harvested by centrifugation. The cell pellets were stored at −80°C until use.

### Purification of the CX3CR1-G_i1_ complex

The cell pellets of 200 ml of cell culture were thawed at 4°C and suspended by Dounce homogenization in the lysis buffer containing 20 mM Hepes (pH 7.5), 50 mM NaCl, 2 mM MgCl_2_, and EDTA-free protease inhibitor (Roche). Then, the suspension was incubated at room temperature for 1 hour with apyrase (25 mU ml^−1^; New England Biolabs). The supernatant was removed by ultracentrifugation at 160,000× *g* for 30 min, and the membrane pellet was resuspended and solubilized in a buffer containing 50 mM Hepes (pH 7.5), 150 mM NaCl, 0.5% (w/v) *n*-dodecyl-β-d-maltopyranoside (DDM; Anatrace), 0.1% (w/v) cholesterol hemisuccinate (CHS; Sigma-Aldrich), EDTA-free protease inhibitor, and apyrase (25 mU ml^−1^). The supernatant was isolated by ultracentrifugation and then incubated with Strep-Tactin Sepharose (IBA Lifesciences) overnight at 4°C. The resin was loaded into a gravity column and washed with 10 column volumes of 25 mM Hepes (pH 7.5), 150 mM NaCl, 0.05% (w/v) DDM, and 0.01% (w/v) CHS. Detergent was exchanged by incubating with exchanging buffer [25 mM Hepes, 150 mM NaCl, 0.8% (w/v) lauryl maltoseneopentyl glycol (LMNG; Anatrace), 0.27% (w/v) glyco-diosgenin (GDN), and 0.08% (w/v) CHS] at 4°C for 2 hours followed by washing with 10 column volumes of 25 mM Hepes (pH 7.5), 150 mM NaCl, 0.01% (w/v) LMNG, 0.0033% (w/v) GDN, and 0.001% (w/v) CHS. The complex protein was eluted with 4 column volumes of elute buffer containing 200 mM tris (pH 8.0), 50 mM biotin, 150 mM NaCl, 0.01% (w/v) LMNG, 0.0033% (w/v) GDN, and 0.001% (w/v) CHS and concentrated to 500 μl with a 100-kDa molecular weight cutoff concentrator (Millipore). The complex protein was further purified by size exclusion chromatography with a pre-equilibrated [25 mM Hepes (pH 7.5), 150 mM NaCl, 0.002% (w/v) LMNG, 0.00067% (w/v) GDN, and 0.0002% (w/v) CHS] Superdex 200 Increase 10/300 column (GE Healthcare); the fraction containing the complex was concentrated to 3 mg ml^−1^. The protein quality was analyzed by analytical size exclusion chromatography, native polyacrylamide gel electrophoresis (PAGE), and SDS-PAGE.

### Purification of the CX3CR1-CX3CL1-G_i1_ complex

The cells expressing the CX3CR1-CX3CL1-G_i1_ complex were thawed on ice and suspended in a buffer containing 20 mM Hepes (pH 7.5), 50 mM NaCl, 2 mM MgCl_2_, and EDTA-free protease inhibitor. After 1 hour of incubation with apyrase (25 mU ml^−1^) at room temperature, the supernatant was removed by ultracentrifugation at 160,000× *g* for 30 min. The protein was extracted by adding solubilization buffer containing 50 mM Hepes (pH 7.5), 150 mM NaCl, 0.5% (w/v) DDM, 0.1% (w/v) CHS, EDTA-free protease inhibitor, and apyrase (25 mU ml^−1^). The mixture was incubated at 4°C for 2 hours. The supernatant was isolated by ultracentrifugation and then incubated overnight with Strep-Tactin Sepharose at 4°C. The resin was washed with 10 column volumes of a buffer containing 25 mM Hepes (pH 7.5), 150 mM NaCl, 0.05% (w/v) DDM, and 0.01% (w/v) CHS and then incubated with a buffer containing 25 mM Hepes (pH 7.5), 150 mM NaCl, 0.1% (w/v) LMNG, and 0.01% (w/v) CHS at 4°C for 2 hours. The resin was washed with 10 column volumes of wash buffer containing 25 mM Hepes (pH 7.5), 150 mM NaCl, 0.01% (w/v) LMNG, and 0.001% (w/v) CHS and then eluted with 4 column volumes of elute buffer containing 200 mM tris (pH 8.0), 50 mM biotin, 150 mM NaCl, 0.01% (w/v) LMNG, and 0.001% (w/v) CHS. The complex protein was further purified by size exclusion chromatography using a Superdex 200 Increase 10/300 column, which was pre-equilibrated with 25 mM Hepes (pH 7.5), 150 mM NaCl, 0.002% (w/v) LMNG, and 0.0002% (w/v) CHS. The purified complex was concentrated to 3 mg ml^−1^ with a 100-kDa molecular weight cutoff concentrator, and the protein quality was analyzed by analytical size exclusion chromatography, native PAGE, and SDS-PAGE.

### Expression of CX3CL1

The coding sequence of CX3CL1 (residues 1 to 77) followed by a PreScission protease site (LEVLFQGP) and green fluorescent protein (GFP) with a flexible linker (SGSGSAAA) was cloned into pEG BacMam vector (Addgene, catalog no. 160451), and a 10 × His-tag was added at the C terminus for purification. CX3CL1-GFP was expressed in human embryonic kidney (HEK) 293F cells with baculovirus transduction. Baculovirus was added to cells at a density of 2.0 × 10^6^ cells ml^−1^. Cells were removed by centrifugation, and the culture supernatant was incubated with Ni-NTA resin (QIAGEN) at 4°C for 2 hours; the CX3CL1-GFP immobilized resin was washed with 10 column volumes of purification buffer containing 25 mM Hepes (pH 7.5) and 150 mM NaCl supplemented with 20 mM imidazole. CX3CL1-GFP was eluted with the purification buffer containing 300 mM imidazole. PD MiniTrap (GE Healthcare) was used to remove imidazole. The protein was then digested with His-tagged PreScission protease (homemade) and His-tagged PNGase F (homemade) overnight to remove the C-terminal GFP-His-tag-Flag-tag and deglycosylate the chemokine. After reverse binding to Ni-NTA resin, the chemokine was further purified and then concentrated to about 2 mg ml^−1^. The final chemokine protein was frozen with liquid nitrogen and stored at −80°C until use. The protein quality was analyzed by Nu-PAGE.

### Disulfide cross-linking

CX3CR1 tagged with 10 × His and Flag at the C terminus and CX3CL1 tagged with GFP-Flag containing respective single-cysteine mutation were co-expressed in HEK293F cells (Invitrogen). The cell pellets were thawed on ice and lysed by repeated Dounce homogenization and centrifugation in a hypotonic buffer containing 10 mM Hepes (pH 7.5), 10 mM MgCl_2_, 20 mM KCl, and EDTA-free protease inhibitor cocktail (Roche), followed by two more washes using a high osmotic buffer containing 10 mM Hepes (pH 7.5), 10 mM MgCl_2_, 20 mM KCl, and 1 M NaCl. The purified membranes were resuspended in 10 mM Hepes (pH 7.5), 10 mM MgCl_2_, 20 mM KCl, 30% (w/v) glycerol, and EDTA-free protease inhibitor cocktail; flash-frozen with liquid nitrogen; and stored at −80°C until use.

The purified membranes were thawed on ice and solubilized in a buffer containing 50 mM Hepes (pH 7.5), 500 mM NaCl, 0.5% (w/v) DDM, and 0.1% CHS at 4°C for 3 hours. After centrifugation at 160,000× *g* for 30 min, the supernatant was incubated with the TALON Superflow Metal Affinity Resin (Clontech) supplemented with 10 mM imidazole at 4°C overnight. The resin was washed with 20 column volumes of wash buffer containing 25 mM Hepes (pH 7.5), 500 mM NaCl, 0.05% (w/v) DDM, 0.01% (w/v) CHS, 10% (w/v) glycerol, and 30 mM imidazole at 4°C. The protein was eluted with 5 column volumes of 25 mM Hepes (pH 7.5), 500 mM NaCl, 0.05% (w/v) DDM, 0.01% (w/v) CHS, 10% (w/v) glycerol, and 300 mM imidazole.

The purified sample was digested with PreScission protease to remove the GFP. Then, the digested sample was analyzed by 10% nonreducing Nu-PAGE and Western blot. Western blot was performed to specifically identify the Flag-tagged CX3CR1 and CX3CR1-CX3CL1 complex. The nitrocellulose membrane was incubated in 50 ml of phosphate-buffered saline (PBS) buffer [137 mM NaCl, 2.7 mM KCl, 10 mM Na_2_HPO_4_, and 2 mM KH_2_PO_4_ (pH 7.4)], supplemented with 5% milk (w/v) and 5 μl of primary antibody [mouse monoclonal anti-Flag antibody (1:2000 diluted in PBS; Sigma-Aldrich, catalog no. F3165)] for 1 hour at room temperature. Then, the nonspecifically bound antibody was washed three times with 10 ml of PBST buffer [PBS buffer supplemented with 0.1% Tween-20 (v/v)] for 10 min, followed by incubation with the secondary antibody [goat anti-mouse IgG (1:2000 diluted in PBS; Sigma-Aldrich, catalog no. AP124)] for 1 hour at room temperature and three washes with PBST. Last, the cross-linked complex was detected by 5-bromo-4-chloro-3-indolyl-phosphate (Sigma-Aldrich, catalog no. 6578-06-9).

### Cryo-EM data collection

The freshly purified CX3CR1-G_i1_ complex was diluted to 2.2 mg ml^−1^ with a buffer containing 25 mM Hepes (pH 7.5) and 150 mM NaCl, and 3 μl of protein sample was applied onto the glow-discharged holey grids (CryoMatrix R1.2/1.3, Au 300 mesh). For the CX3CR1-CX3CL1-G_i1_ complex, the working concentration was 1.0 mg ml^−1^, and the protein was also vitrified in holey grids (CryoMatrix R1.2/1.3, Au 300 mesh). The grids were then blotted at 4°C and 100% humidity with a blot force of 0 and vitrified with liquid ethane cooled by liquid nitrogen using a Mark IV Vitrobot (Thermo Fisher Scientific). Cryo-EM images were collected on a 300-kV Titan Krios G3 electron microscope (FEI) equipped with a Gatan K3 summit direction camera and a Gatan Quantum LS imaging filter (GIF, Gatan) with a slit width of 20 eV. The superresolution counting mode at a magnification of ×81,000 was used to capture movies automatically with a pixel size of 1.045 Å. Movie stacks were recorded with defocus values varying from −0.8 to 1.5 μm and generated by 3-s exposure with 32 frames.

### Cryo-EM data processing

A total of 9076 movies for the CX3CR1-G_i1_ complex and 8604 movies for the CX3CR1-CX3CL1-G_i1_ complex were collected and subjected to a beam-induced motion correction using MotionCor2 (*42*). In addition, the contrast transfer function for each image was estimated using the Gctf software (*43*). Particle selection, initial model generation, two-dimensional (2D) classification, 3D classification, and 3D refinement were carried out in REILON 3.0 (*44*). A total of 4,846,904 particles of the CX3CR1-G_i1_ complex and 3,394,697 particles of the CX3CR1-CX3CL1-G_i1_ complex were extracted for 2D classification and 3D classification. For the CX3CR1-G_i1_ complex, a subset of 702,722 particles, which appeared to be the best-looking ones, was subjected to 3D refinement and Bayesian polishing using REILON 3.0, yielding a final map with a global resolution of 2.8 Å. For the CX3CR1-CX3CL1-G_i1_ complex, 490,779 particles selected were subjected to 3D refinement and Bayesian polishing and yielded a final map with a global resolution of 3.4 Å. Both reported resolutions were determined on the basis of the gold standard Fourier shell correlation using the 0.143 criterion. Local resolution was determined using ResMap (*45*).

### Model building and refinement

The crystal structure of CX3CL1-bound US28 (PDB ID: 4XT1) and G_i1_ from the μ-opioid receptor (μOR)–G_i_ structure (PDB ID: 6DDE) was taken as the initial models for both CX3CR1-G_i1_ and CX3CR1-CX3CL1-G_i1_ complexes (table S1). Model fitting was accomplished using Chimera (University of California San Francisco, UCSF) (*46*). The sequence was replaced with CX3CR1 and iterative manual adjustment was performed in Coot (*47*). The final model of CX3CR1-G_i1_ contains 288 residues of CX3CR1 (I23 to Y310). The final model of CX3CR1-CX3CL1-G_i1_ contains 287 residues of CX3CR1 (Y22 to Y308) as well as pE1 to C50 and E55 to D66 of CX3CL1. The remaining residues of CX3CR1 and CX3CL1 are disordered and were not modeled. After rounds of global refinement, real-space refinement was carried out by Phenix (*48*). The models were validated using MolProbity. Structural figures in the manuscript were prepared using Chimera/Chimera.X and PyMOL (https://pymol.org/2/).

### IP accumulation assay

Flag-tagged WT and mutant CX3CR1 were cloned into the pTT5 vector (Invitrogen). CX3CR1 and a chimeric Gα protein Gα_qi5_ (the last five C-terminal residues of Gα_q_ were replaced with those in Gα_i_) were cotransfected into HEK293F cells (Invitrogen, catalog no. K1663) at a plasmid ratio of 1:2 (w/w). Cell surface expression was measured 48 hours after transfection by mixing 10 μl of cells and 15 μl of monoclonal ANTI-FLAG M2-FITC antibody [1:100 diluted by tris-buffered saline supplemented with 4% bovine serum albumin (BSA); Sigma-Aldrich, catalog no. F4049]. After 20 min, the fluorescence signal on the cell surface was measured by a flow cytometry reader (Guava easyCyte HT, Millipore).

IP accumulation was measured using an HTRF IP-One G_q_ kit (Cisbio Bioassays, 62IPAPEB), and the experiment was carried out following the manufacturer’s instructions. In brief, 7 μl of transfected HEK293F cells was seeded in a 384-well plate (20,000 cells per well) and then 7 μl of CX3CL1 (different concentrations diluted in stimulation buffer) was added and incubated at 37°C for 2 hours. Three microliters of d2-labeled IP1 and 3 μl of cryptate-labeled anti-IP1 monoclonal antibody, which was prediluted in lysis buffer (1:20), were added into the plate and incubated at room temperature for 1 hour. Plates were read in Synergy H1 Operator (BioTek) with excitation at 330 nm and emission at 620 and 665 nm. Data were analyzed using GraphPad Prism 8.0. EC_50_ and pEC_50_ ± SEM were calculated using nonlinear regression (curve fit).

### TRUPATH assay

The plasmids of Gα_i1_-Rluc8, Gβ_3_, Gγ_9_-GFP2, and CX3CR1 were cotransfected into 2 ml of HEK293F cells at a plasmid ratio of 1:1:1:2 (w/w) with a total amount of 4 μg. Cell surface expression was measured as previously described in IP accumulation assay. Sixty microliters of cells suspended in BRET2 assay buffer [1 × Hanks’ balanced salt solution (Gibco), 20 mM Hepes (pH 7.4), and 0.01% BSA] was seeded in a 96-well plate (20,000 cells per well), and the plates were placed in 37°C for 30 min to adapt to the buffer environment. Then, 10 μl of freshly prepared 50 μM coelenterazine 400a (Nanolight Technologies) was added into the 96-well plate. After a 5-min equilibration period, the plates were then read in a plate reader (BioTek) for 10 min with 395-nm (RLuc8-coelenterazine 400a) and 510-nm (GFP2) emission filters, at integration times of 3 s per well. Cells were then treated with 30 μl of ligand [CX3CL1 (homemade), CCL3 (PeproTech, catalog no. AF-300-08)] with different concentrations diluted in the assay buffer, and signals were detected immediately for 20 min. BRET2 ratios were calculated as the ratio of the GFP2 emission to RLuc8 emission, and data were analyzed using GraphPad Prism 8.0.

## Notes

View/request a protocol for this paper from *Bio-protocol*.
