# Supplementary material for: Activation of the human chemokine receptor CX3CR1 regulated by cholesterol
Source: Sci Adv. 2022 Jun 29;8(26):eabn8048. doi: 10.1126/sciadv.abn8048 (PMC9242592; doi:10.1126/sciadv.abn8048)
Supplement: Supplementary file 1 — Figs. S1 to S6 Tables S1 to S5 [file sciadv.abn8048_sm.pdf]

Science Advances
AAAS

Supplementary Materials for

Activation of the human chemokine receptor CX3CR1 regulated by cholesterol

Minmin Lu *et al.*

Corresponding author: Ya Zhu, zhuya2@simm.ac.cn; Beili Wu, beiliwu@simm.ac.cn; Qiang Zhao, zhaoq@simm.ac.cn

Sci. Adv. **8**, eabn8048 (2022)
DOI: 10.1126/sciadv.abn8048

This PDF file includes:

Figs. S1 to S6
Tables S1 to S5

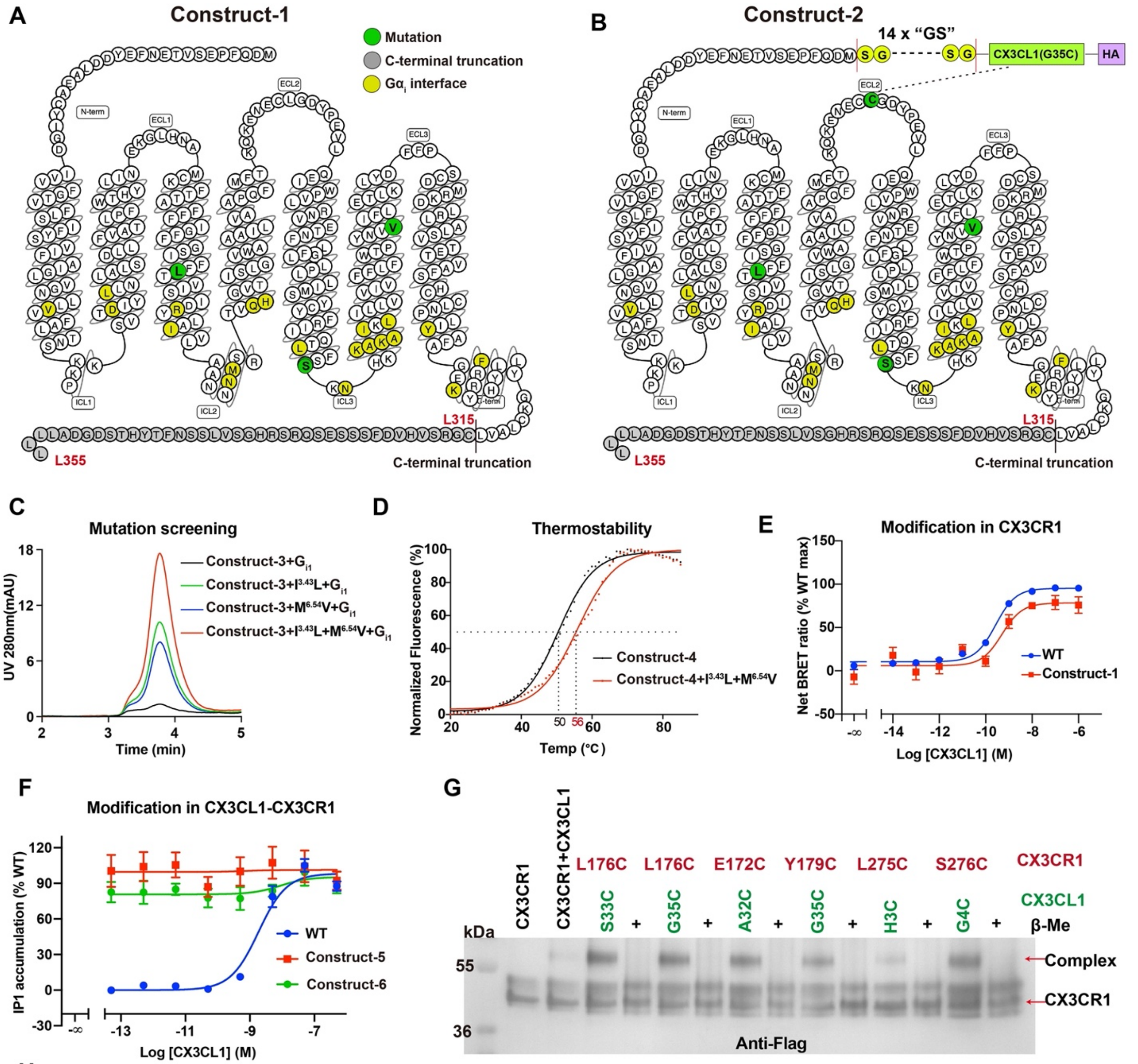

H

Construct	Mutation	Truncation	Linker between CX3CL1 and CX3CR1	Disulfide crosslinking between CX3CL1 and CX3CR1	Fusion
Construct-1	C221S, I$^{3.43}$L, M$^{6.54}$V	ΔC316-L355	/	/	/
Construct-2	C221S, I$^{3.43}$L, M$^{6.54}$V	ΔC316-L355	14 x GS	CX3CR1 (L176C)-CX3CL1 (G35C)	/
Construct-3	C221S	ΔC316-L355	/	/	/
Construct-4	C221S,K$^{6.30}$E	ΔC316-L355	/	/	N-Bril
Construct-5	C221S, I$^{3.43}$L, M$^{6.54}$V	ΔC316-L355	7 x GS-G-Flag-3 x GS	CX3CR1 (L176C)-CX3CL1 (G35C)	/
Construct-6	C221S, I$^{3.43}$L, M$^{6.54}$V	ΔC316-L355	7 x GS-G-Flag-3 x GS	/	/

Fig. S1. Optimization and characterization of CX3CR1 constructs. (**A** and **B**) Construct information of CX3CR1 used for CX3CR1-G$_{i1}$ (A) and CX3CL1-CX3CR1-G$_{i1}$ (B) structure determination. The mutated, truncated and involved in G protein interface residues are shown as

green, dark gray and dark yellow solid circles, respectively. (**C**) Analytical size-exclusion chromatography (aSEC) result of purified CX3CR1-G_{i1} complex with different CX3CR1 mutants. (**D**) Thermostability assay of CX3CR1 mutants. (**E**) CX3CL1-induced G protein activation of the WT and modified CX3CR1. At least three independent experiments (n) were performed in triplicate, data are shown as mean ± SEM. See table S3 for detailed statistical evaluation. (**F**) CX3CL1-induced IP accumulation of the WT CX3CR1 and the fused form of CX3CL1-CX3CR1 with or without disulfide crosslinking mutations [CX3CL1 (G35C)-CX3CR1 (L176C)]. At least three independent experiments (n) were performed in triplicate, data are shown as mean ± SEM. See table S2 for detailed statistical evaluation. (**G**) The result of anti-Flag western blot in disulfide bond crosslinking screening of CX3CL1 and CX3CR1. The western blot was performed with mouse anti-Flag antibody and polyclonal goat anti-mouse IgG antibody to specifically identify Flag-tagged CX3CR1. The red arrows indicate the bands of CX3CL1-CX3CR1 complex and CX3CR1. (**H**) Modifications of constructs used in structure determination and optimization. Construct 1, the CX3CR1 construct used to determine the CX3CR1-G_{i1} structure; construct 2, the CX3CR1 construct used to determine the CX3CL1-CX3CR1-G_{i1} structure; construct 3 and construct 4 are the CX3CR1 constructs used in construct optimization; construct 5 and construct 6 are the fused form of CX3CL1-CX3CR1 with and without disulfide crosslinking mutations respectively, and are used to validate the effect of "14 × Gly-Ser" linker and disulfide bond (CX3CL1 (G35C)-CX3CR1 (L176C)).

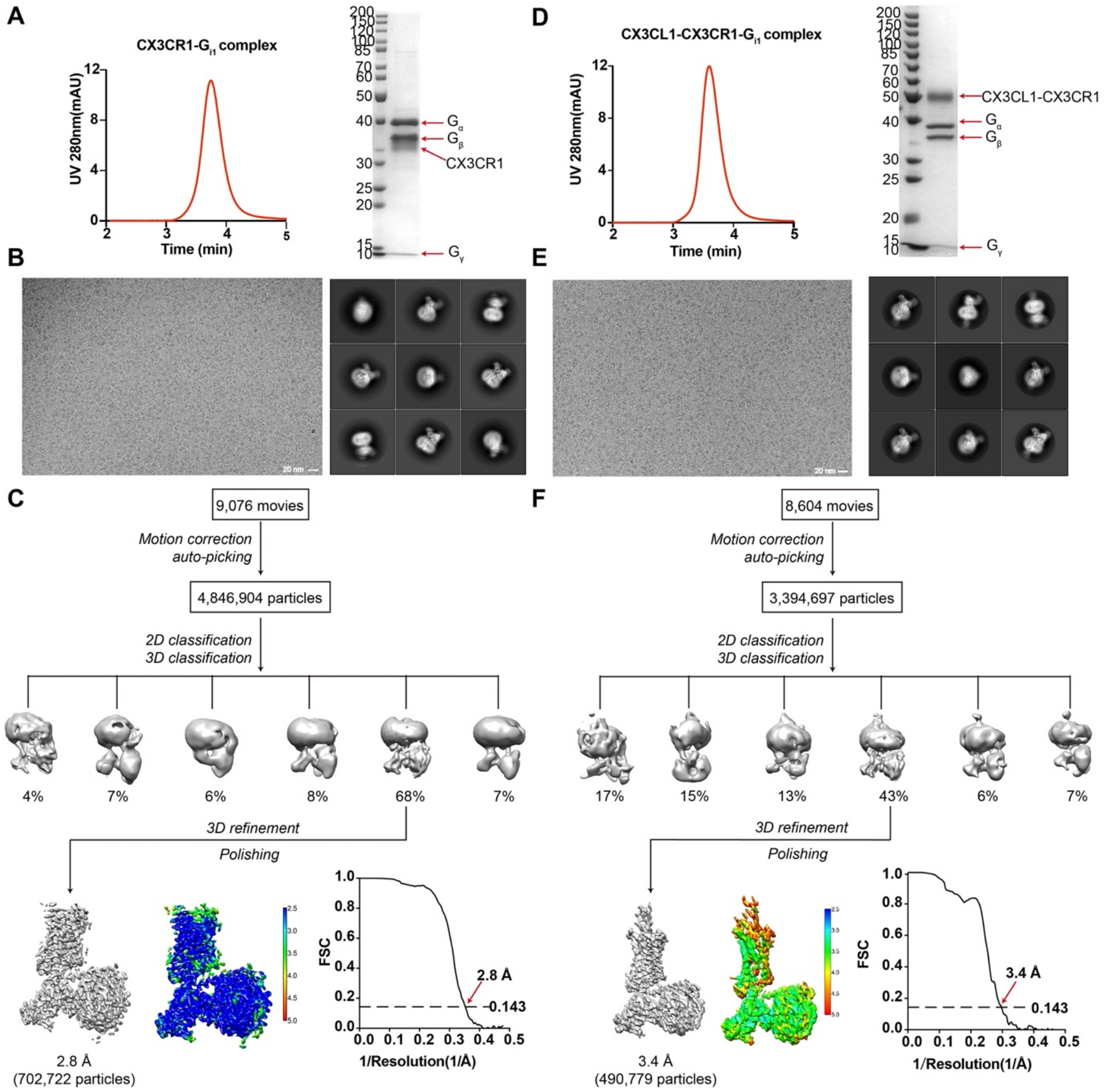

Fig. S2. Sample preparation and cryo-EM data processing of G_{i1}-bound CX3CR1 complexes. **(A-C)** Results of CX3CR1-G_{i1} complex. **(A)** aSEC and SDS-PAGE results of CX3CR1-G_{i1} complex. **(B)** Representative cryo-EM image and 2D average of CX3CR1-G_{i1} complex. **(C)** Workflow of cryo-EM data processing with cryo-EM map colored according to local resolution (Å). Gold-standard FSC curve showing an overall resolution at 2.8 Å (red arrow) for CX3CR1-G_{i1}. (**D-F**) Results of CX3CL1-CX3CR1-G_{i1} complex. **(D)** aSEC and SDS-PAGE results of CX3CL1-CX3CR1-G_{i1} complex. **(E)** Representative cryo-EM image and 2D average of CX3CL1-

CX3CR1-G_{i1} complex. **(F)** Workflow of cryo-EM data processing with cryo-EM map colored according to local resolution (Å). Gold-standard FSC curve showing an overall resolution at 3.4 Å (red arrow) for CX3CL1-CX3CR1-G_{i1}.

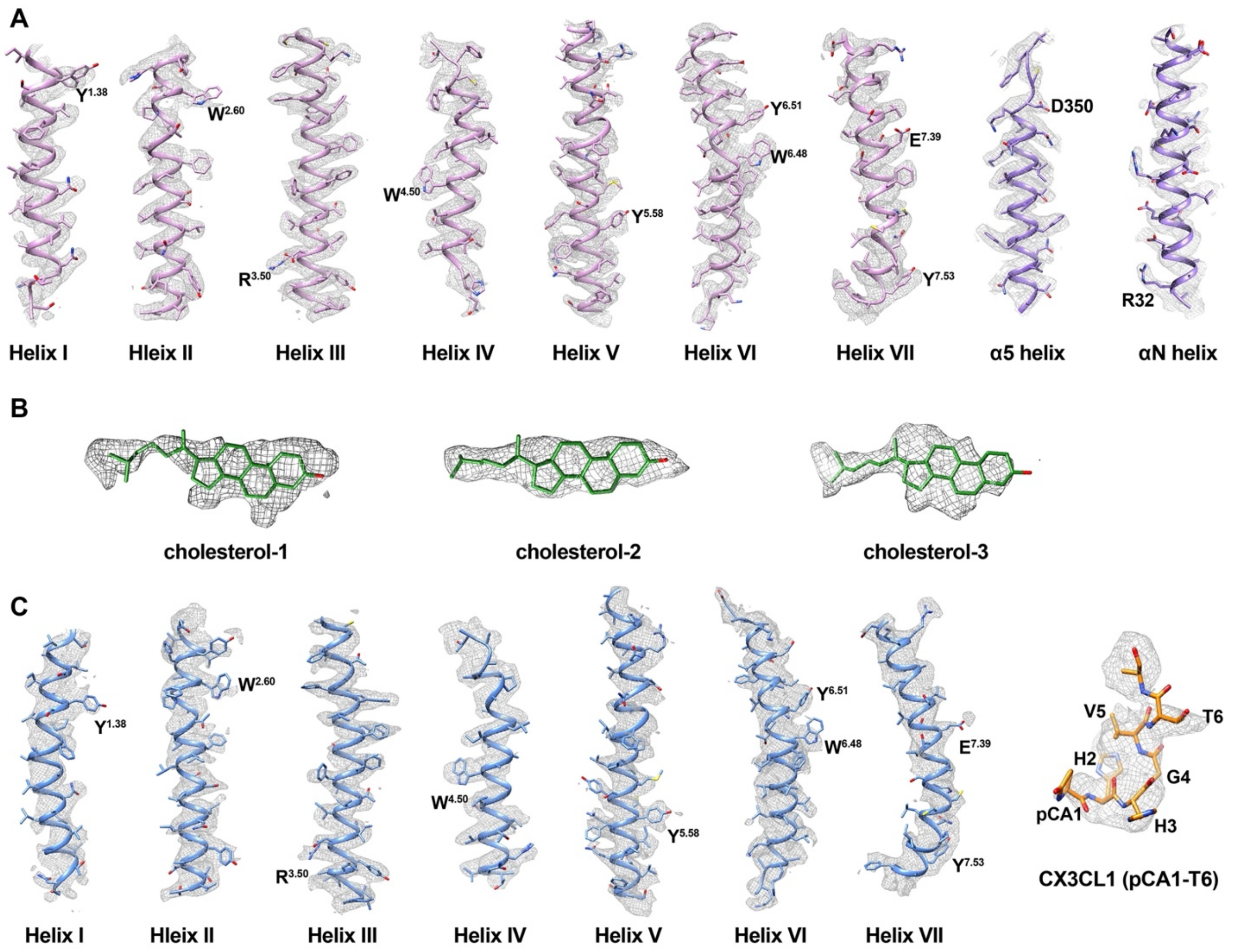

Fig. S3. Electron density maps of the CX3CR1 structures. (A) Cryo-EM density map and model of all transmembrane helices of CX3CR1, α5- and αN-helixes of $G\alpha_{i1}$ in CX3CR1-G_{i1} complex. **(B)** Cryo-EM density map and model of cholesterol molecules. **(C)** Cryo-EM density map and model of the CX3CL1-CX3CR1-G_{i1} complex structure are displayed for all transmembrane helices, and residues (pE1-T6) of CX3CL1.

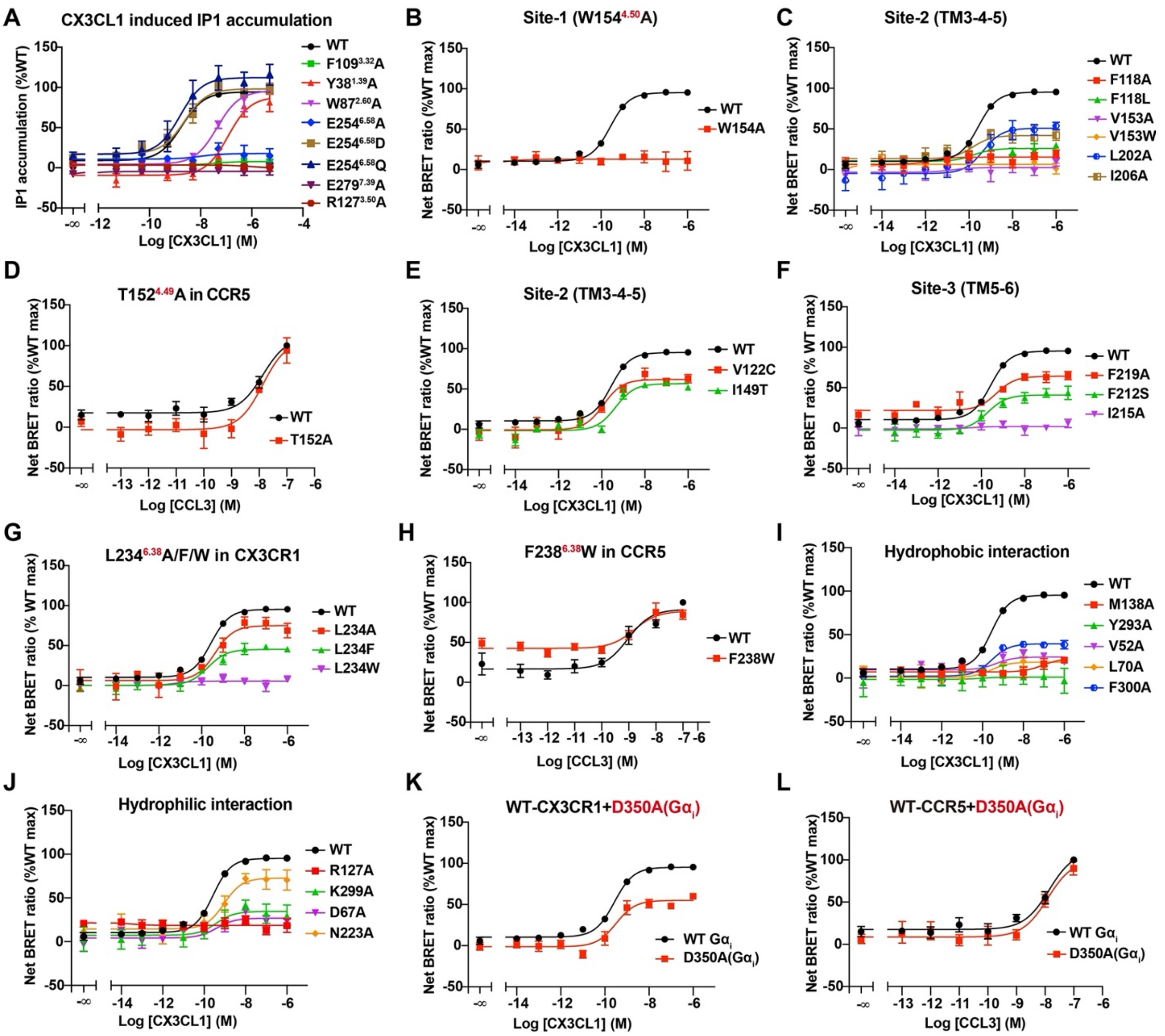

Fig. S4. Signaling assays of CX3CR1 and CCR5. (A) CX3CL1-induced IP accumulation of WT and mutant CX3CR1. **(B)** CX3CL1-induced G protein activation tested by TRUPATH sensor, reflecting the effects of W154$^{4.50}$A. **(C and E)** CX3CL1-induced G protein activation of WT and mutant CX3CR1 in site-2 tested by TRUPATH sensor, reflecting the effects of receptor mutants in site-2. **(D)** CCL3-induced G protein activation of WT CCR5 and T152$^{4.49}$A. **(F-G)** CX3CL1-induced G protein activation of CX3CR1 tested by TRUPATH sensor, reflecting the effects of receptor mutants in site-3. **(H)** CCL3-induced G protein activation of WT CCR5 and F238$^{6.38}$W. **(I-J)** CX3CR1-induced G protein activation of WT and mutant CX3CR1. (**K**) CX3CL1-induced

G protein activation of WT CX3CR1 using the mutant $G\alpha_i$ (D350A). **(L)** CCL3-induced G protein activation of WT CCR5 using the mutant $G\alpha_i$ (D350A). At least three independent experiments (n) were performed in triplicate, data are shown as mean ± SEM. See table S2, S3 and S4 for detailed statistical evaluation.

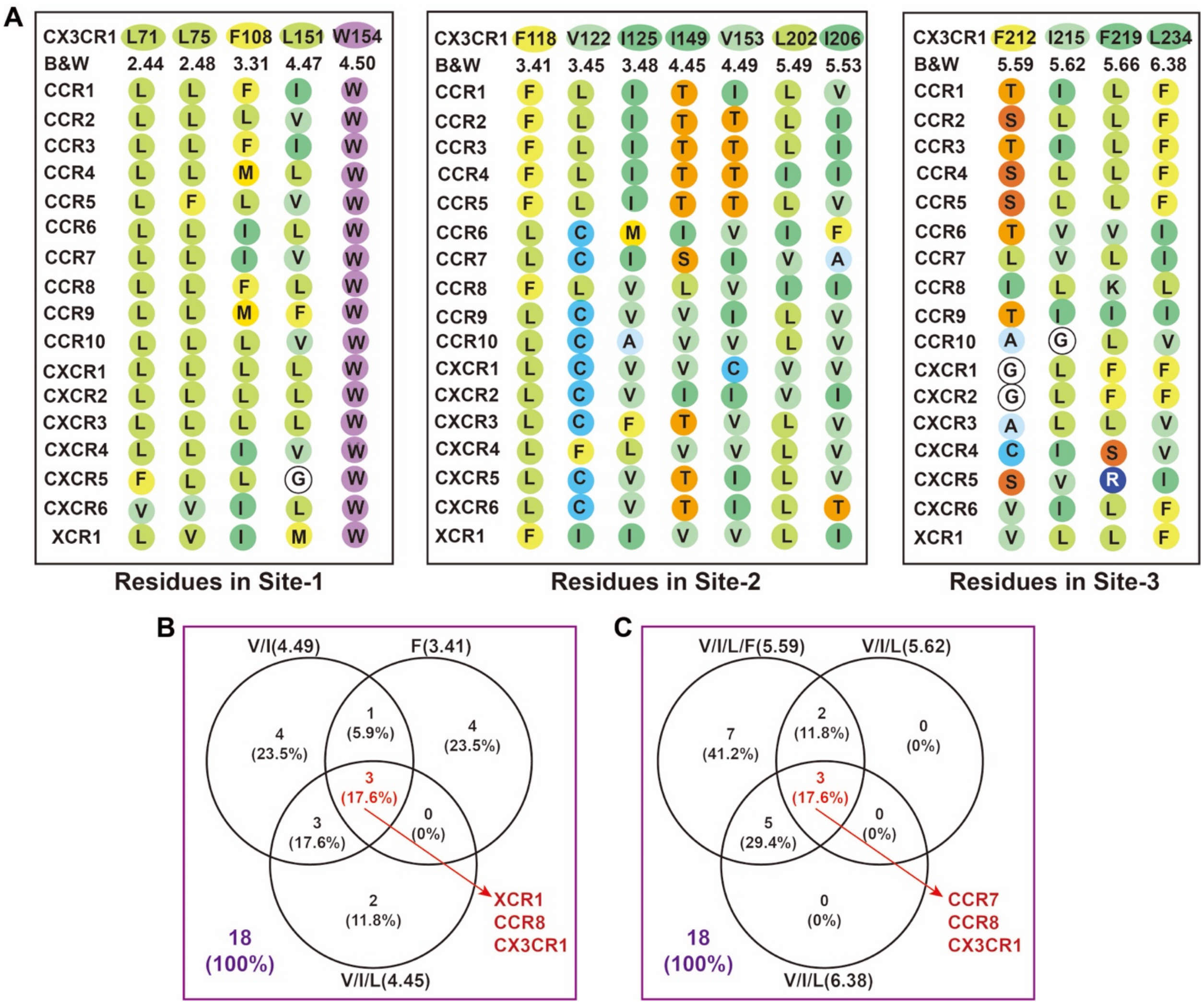

Fig. S5. Sequence alignment of equivalent residues in CX3CR1 cholesterol binding pockets of human CKRs. (A) Sequence alignment results of cholesterol binding pockets in human CKRs. Different residues are labeled with distinct colors. **(B-C)** Venn diagrams illustrating the abundance of key elements involved in binding at site-2 and site-3 among CKRs.

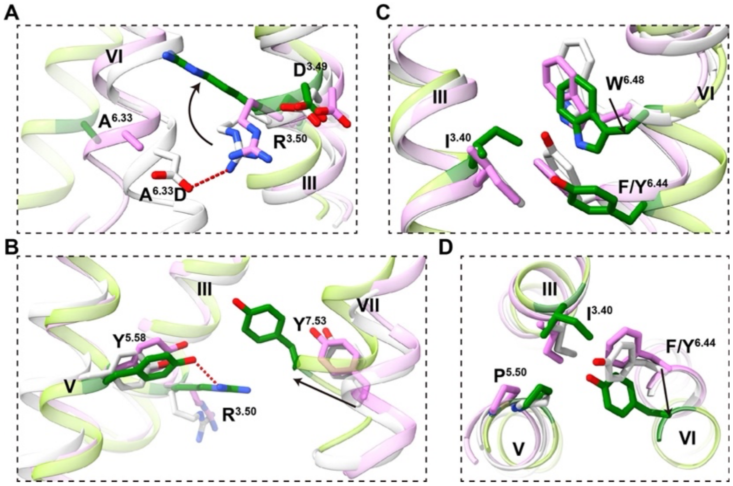

Fig. S6. Structural comparison of conserved motifs in CX3CR1 and CCR5. (A-D) Superposition of the inactive CCR5 (PDB ID: 4MBS), active CCR5 (PDB ID: 7F1Q) and active CX3CR1, which are shown in gray, green and pink with cartoon representations, respectively. Residues in each motif are shown as sticks. The red dashed line shows the salt bridge. The black arrows indicate conformational changes of conserved motifs between inactive and active CCR5. **(A)** Conformational changes of "DRY" motif. **(B)** Rearrangement of $Y^{7.53}$ in "NPxxY" motif. **(C)** Conformational changes of "toggle switch"-$W^{6.48}$. **(D)** Rearrangement of "PIF" motif.

Table S1. Cryo-EM data collection and refinement statistics of CX3CR1-G_{i1} and CX3CL1-CX3CR1-G_{i1} complex structures.

	**CX3CR1-G_{i1}** (PDB-7XBW) (EMDB-33107)	**CX3CL1-CX3CR1-G_{i1}** (PDB-7XBX) (EMDB-33108)
**Data collection and processing**		
Magnification	81,000	81,000
Voltage (kV)	300	300
Electron exposure (e-/Å^2)	70	70
Defocus range (μm)	-0.8 ~ -1.5	-0.8 ~ -1.5
Pixel size (Å)	1.045	1.045
Symmetry imposed	C1	C1
Initial particle images (no.)	4,846,904	3,394,697
Final particle images (no.)	702,722	490,779
Map resolution (Å)	2.8	3.4
FSC threshold	0.143	0.143
Map resolution range (Å)	2.5-5.0	2.5-5.0
**Refinement**		
Initial model used (PDB code)	6DDE,4XT1	6DDE, 4XT1
Model resolution (Å)	3.0	4.1
Map sharpening B factor (Å^2)	-101	-128
Model composition		
Receptor residues	288 (2,260 atoms)	287 (2,119 atoms)
Chemokine residues	/	61 (402 atoms)
G_{i1} residues	598 (4,405 atoms)	560 (4,087 atoms)
B-factors (Å^2)		
Receptor	70.41	151.50
Chemokine	/	115.15
G_{i1}	43.99	75.71
R.m.s. deviations		
Bond lengths (Å)	0.002	0.002
Bond angles (°)	0.494	0.430
Validation		
MolProbity score<br>Clash score<br>Poor rotamers (%)	1.40<br>6.14<br>0.0	1.43<br>6.35<br>0.0
Ramachandran plot		
Favored (%)<br>Allowed (%)<br>Disallowed (%)	97.71<br>2.29<br>0.0	97.65<br>2.35<br>0.0

Table S2. CX3CL1-induced IP accumulation of WT and mutant CX3CR1 using a chimeric Gα protein $G\alpha_{qi5}$.

	CX3CL1-induced IP accumulation					
Mutants	EC_{50} (nM)	Ratio[a]	pEC_{50} ± SEM[b]	Span[b,c] (% of WT)	n[d]	Expression[e] (% ofWT) Mean± SEM
WT	1.82	1	8.74 ± 0.08	100 ± 3	14	100
$Y38^{1.39}A$[f]	119.84	66	6.92 ± 0.19*	114 ± 10	5	104 ± 21
$W87^{2.60}A$	44.67	25	7.35 ± 0.13	107 ± 6	3	153 ± 9
$F109^{3.32}A$	ND	ND	ND	ND	4	106 ± 21
$E254^{5.38}A$	ND	ND	ND	ND	3	60 ± 17
$E254^{5.38}D$	2.32	1	8.64 ± 0.18	95 ± 7	5	164 ± 16
$E254^{5.38}Q$	1.56	0.8	8.81 ± 0.26	112 ± 11	3	75 ± 10
$E279^{7.39}A$	ND	ND	ND	ND	4	93 ± 16
$R127^{3.50}A$	ND	ND	ND	ND	4	96 ± 21
Construct-5[g]	ND	ND	ND	ND	6	304 ± 8***
Construct-6[h]	8.9	4.9	8.05 ± 1.18	15 ± 8***	5	169 ± 11

[a]The EC50 ratio, $EC_{50(mutant)}/EC_{50(WT)}$, refers to the shift between the WT CX3CR1 and mutant curves, characterizing the effect of the mutations on receptor signaling.

[b]Data are shown as mean ± SEM from at least three independent experiment each performed in technical triplicate. *P<0.01; **P<0.001; ***P<0.0001 by one-way ANOVA followed by Dunnett's post-test, compared with the response of the WT.

[c]The span is defined as the window between the maximal CX3CL1 response (E_{max}) and the vehicle (no ligand). "ND" (not determined) refers to data where a robust concentration response curve could not be established within the concentration range tested, such that an E_{max} was not reached and therefore span could not be calculated.

[d]Sample size: the number of independent experiments performed in triplicate.

[e]Protein expression levels of CX3CR1 constructs at the cell surface were determined in parallel by flow cytometry with an anti-FLAG antibody and reported as per cent compared to the WT form three independent measurements performed in duplicate.

[f]All mutations were introduced in the WT CX3CR1.

[g]The construct used to validate the effect of "14 × Gly-Ser" linker and disulfide bond between CX3CL1 (G35C) and CX3CR1 (L176C).

[h]The construct used to validate the effect of "14 × Gly-Ser" linker between CX3CL1 and CX3CR1.

Table S3. CX3CL1-induced TRUPATH assay of WT and mutant CX3CR1.

CX3CL1-induced TRUPATH assay of CX3CR1						
Mutants	EC_{50} (nM)	Ratio[a]	pEC_{50} ± SEM[b]	Span[b,c] (% of WT)	n[d]	Expression[e] (% of WT) Mean ± SEM
WT	0.27	1	9.57 ± 0.07	100 ± 2	25	100
Construct-1[f]	0.17	0.6	9.76 ± 0.23	80 ± 6*	5	123 ± 17
$F118^{3.41}A$[g]	ND	ND	ND	ND	4	86 ± 20
$F118^{3.41}L$	0.16	0.6	9.79 ± 0.46	19 ± 3***	4	115 ± 12
$V122^{3.45}C$	0.12	0.4	9.91 ± 0.24	74 ± 7**	4	142 ± 24
$I149^{4.45}T$	0.45	2	9.35 ± 0.19	69 ± 5***	4	154 ± 46
$V153^{4.49}A$	ND	ND	ND	ND	4	216 ± 21***
$V153^{4.49}W$	ND	ND	ND	ND	4	73 ± 3
$L202^{5.49}A$	0.45	2	9.35 ± 0.35	64 ± 5***	3	226 ± 15***
$I206^{5.53}A$	0.10	0.4	9.98 ± 0.26	37 ± 4***	4	101 ± 2
$F212^{5.59}S$	0.19	1	9.71 ± 0.27	51 ± 5***	3	130 ± 28
$I215^{5.62}A$	ND	ND	ND	ND	3	64 ± 11
$F219^{5.66}A$	0.50	2	9.30 ± 0.23	50 ± 4***	4	267 ± 28***
$L234^{6.38}A$	0.27	1	9.37 ± 0.26	81 ± 8*	4	185 ± 21***
$L234^{6.38}F$	0.20	0.7	9.71 ± 0.23	53 ± 5***	3	144 ± 21
$L234^{6.38}W$	ND	ND	ND	ND	5	56 ± 8
$W154^{4.50}A$	ND	ND	ND	ND	3	84±13
$M138^{34.54}A$	83.30	309	7.08 ± 0.57***	18 ± 6***	3	462 ± 28***
$Y293^{7.53}A$	ND	ND	ND	ND	4	121 ± 10
$V52^{1.53}A$	0.48	2	9.31 ± 0.58	18 ± 4***	3	99 ± 20
$L70^{2.43}A$	0.28	1	9.56 ± 0.65	21 ± 4***	3	80 ± 14
$F300^{8.50}A$	0.28	1	9.55 ± 0.19	44 ± 3***	3	76 ± 8
$N223^{ICL3}A$	0.93	3	9.03 ± 0.24	69 ± 7***	4	157 ± 16
$R127^{3.50}A$	ND	ND	ND	ND	3	104 ± 8
$K299^{8.49}A$	0.37	1	9.43 ± 0.38	33 ± 4***	3	127 ± 16
$D67^{2.40}A$	0.40	1	9.39 ± 0.49	27 ± 5***	3	134 ± 13
D350A ($G\alpha_i$)[h]	0.32	1	9.50 ± 0.20	66 ± 5***	5	102 ± 10

[a]The EC50 ratio, $EC_{50(mutant)}/EC_{50(WT)}$, refers to the shift between the WT CX3CR1 and mutant curves, characterizing the effect of the mutations on receptor signaling.

[b]Data are shown as mean ± SEM from at least three independent experiment each performed in technical triplicate. *P<0.01; **P<0.001; ***P<0.0001 by one-way ANOVA followed by Dunnett's post-test, compared with the response of the WT.

[c]The span is defined as the window between the maximal CX3CL1 response (E_{max}) and the vehicle (no ligand). "ND" (not determined) refers to data where a robust concentration response curve could not be established within the concentration range tested, such that an E_{max} was not reached and therefore span could not be calculated.

[d]Sample size: the number of independent experiments performed in triplicate.

[e]Protein expression levels of CX3CR1 constructs at the cell surface were determined in parallel by flow cytometry with an anti-FLAG antibody and reported as per cent compared to the WT form three independent measurements performed in duplicate.

[f]The construct used for complex formation with G_{i1}, containing the mutations $I120^{3.43}L$, $C221^{ICL3}S$ and $M250^{6.54}V$ and 40 residues truncated at C terminus.

[g]All mutations were introduced in the WT CX3CR1.

[h]D350A was introduced into $G\alpha_{i1}$-Rluc8.

Table S4. CCL3-induced TRUPATH assay of WT and mutant CCR5.

CCL3-induced TRUPATH assay of CCR5-1[a]						
Mutants	EC_{50} (nM)	Ratio[b]	pEC_{50} ± SEM[c]	Span[c,d] (% of WT)	n[e]	Expression[f] (% of WT) Mean ± SEM
WT	0.93	1	9.03 ± 0.21	100 ± 10	4	100
F238$^{6.38}$W[g]	1.63	2	8.79 ± 0.29	62 ± 8*	4	65 ± 1

CCL3-induced TRUPATH assay of CCR5-2[a]						
Mutants	EC_{50} (nM)	Ratio[b]	pEC_{50} ± SEM[h]	Span[h,d] (% of WT)	n[e]	Expression[f] (% of WT) Mean ± SEM
WT	13.68	1	7.86 ± 0.15	100 ± 10	4	100
T152$^{4.49}$A[g]	13.68	1	7.86 ± 0.25	119 ± 17	3	124 ± 4
D350A ($G\alpha_i$)[i]	12.35	1	7.91 ± 0.22	98 ± 13	4	122 ± 10

[a]Assays of CCR5-1 and CCR-2 were carried out in totally different batch of cells, ligand and assay buffer. All the results of mutant were compared to the corresponding WT.

[b]The EC_{50} ratio, $EC_{50(mutant)}/EC_{50(WT)}$, refers to the shift between the WT CCR5 and mutant curves, characterizing the effect of the mutations on receptor signaling.

[c]Data are shown as mean ± SEM from at least three independent experiment each performed in technical triplicate. *P<0.05 by unpaired t-test, compared with the response of the WT.

[d]The span is defined as the window between the maximal CCL3 response (E_{max}) and the vehicle (no ligand). "ND" (not determined) refers to data where a robust concentration response curve could not be established within the concentration range tested, such that an E_{max} was not reached and therefore span could not be calculated.

[e]Sample size: the number of independent experiments performed in triplicate.

[f]Protein expression levels of CCR5 constructs at the cell surface were determined in parallel by flow cytometry with an anti-FLAG antibody and reported as per cent compared to the WT form three independent measurements performed in duplicate.

[g]F238$^{6.38}$W and T152$^{4.49}$A were introduced in the WT CCR5.

[h]Data are shown as mean ± SEM from at least three independent experiment each performed in technical triplicate. *P<0.01; **P<0.001; ***P<0.0001 by one-way ANOVA followed by Dunnett's post-test, compared with the response of the WT.

[i]D350A was introduced into $G\alpha_{i1}$-Rluc8.

Table S5. DNA and primer sequences of CX3CR1, CCR5 and DNGα_i.

CX3CR1-WT DNA sequence	
ATGGATCAATTCCCAGAGAGCGTTACGGAGAACTTCGAGTACGACGACTTGGCAGAGGCTTGCTACATCGGTGA<br>CATTGTGGTCTTCGGTACCGTGTTCCTGAGCATCTTCTACTCTGTGATCTTCGCTATTGGTTTGGTCGGCAACCTGC<br>TCGTGGTCTTCGCTCTGACAAACTCAAAGAAACCTAAGAGTGTTACGGACATCTACTTGCTGAACCTCGCCTTGT<br>CAGATCTCTTGTTCGTCGCAACACTGCCCTTCTGGACGCACTACCTCATCAACGAGAAGGGCTTGCATAACGCGA<br>TGTGCAAATTCACCACTGCTTTCTTCTTCATTGGATTCTTCGGTTCGATCTTCTTCATTACTGTCATCTCCATTGACA<br>GGTACTTGGCTATCGTTCTGGCTGCCAACAGCATGAACAACAGAACCGTGCAGCACGGCGTCACTATCTCTCTGG<br>GAGTTTGGGCAGCGGCTATTCTCGTGGCCGCACCACAGTTCATGTTCACAAAGCAAAAAGAGAACGAATGTCTG<br>GGCGATTACCCTGAGGTGCTCCAGGAAATCTGGCCCGTTTTGCGCAACGTGGAGACAAACTTCCTGGGATTCCTG<br>CTCCCGTTGCTGATCATGTCGTACTGCTACTTCCGTATCATTCAAACGCTCTTCTCCTGTAAGAACCACAAGAAAG<br>CGAAGGCTATCAAACTCATTCTCTTGGTTGTGATCGTGTTCTTCTTGTTCTGGACCCCATACAACGTCATGATTTTC<br>CTGGAAACTCTGAAGCTCTACGACTTCTTCCCGTCATGCGACATGCGCAAGGATTTGCGTCTGGCTCTCAGCGTC<br>ACAGAGACGGTTGCCTTCTCTCATTGCTGTTTGAACCCACTGATCTACGCCTTCGCAGGTGAAAAGTTCCGCCGT<br>TACCTCTACCACTTGTACGGTAAATGCCTGGCCGTGCTCTGTGGCAGGAGTGTCCATGTTGACTTCTCCAGCTCTG<br>AATCGCAAAGGTCCAGACACGGATCAGTCCTGTCAAGTAACTTCACCTACCATACTAGTGACGGTGACGCTCTGC<br>TCTTGCTG	
WT-F	GTGTTCGCCGGCGCGCCGATGGATCAATTCCCAGAGAGC
C-truncation-R	CAGGAATTCGAGCACGGCCAGGCATTTACCGTACAAGTG
Y38A-R	GAAGATCACAGACGCGAAGATGCTCAGGAACAC
W87A-R	GAGGTAGTGCGTCGCGAAGGGCAGTGTTGCGAC
F109A-R	GAATCCAATGAACGCGAAAGCAGTGGTGAATTT
E254A-R	GAGCTTCAGAGTCGCCAGGAAAATCATGACGTT
E254D-R	GAGCTTCAGAGTGTCCAGGAAAATCATGACGTT
E254Q-R	GTCGTAGAGCTTCAGAGTTTGCAGGAAAATCATGAC
E279A-R	GAAGGCAACCGTCGCTGTGACGCTGAGAGCCAG
R127A-R	GATAGCCAAGTACGCGTCAATGGAGATGACAGT
F118A-R	GGAGATGACAGTAATGAAAGCGATCGAACCGAAGAA
F118L-R	GATGACAGTAATGAACAGGATCGAACCGAAGAATCC
V122C-R	GTACCTGTCAATGGAGATAGCAGTAATGAAGAAGAT
I149T-R	CCAAACTCCCAGAGAGGTAGTGACGCCGTGCTGCAC
V153A-R	GAGAATAGCCGCTGCCCAAGCTCCCAGAGAGATAGT
V153W-R	GAGAATAGCCGCTGCCCACCATCCCAGAGAGATAGT
L202A-R	CGACATGATCAGCAACGGAGCCAGGAATCCCAGGAA
I206A-R	GAAGTAGCAGTACGACATAGCCAGCAACGGGAGCAG
F212A-R	GAGCGTTTGAATGATACGTGCGTAGCAGTACGACAT
I215A-R	ACAGGAGAAGAGCGTTTGAGCGATACGGAAGTAGCA
F219A-R	CTTGTGGTTCTTACAGGAAGCGAGCGTTTGAATGAT
L234A-R	GAACACGATCACAACCAAAGCAATGAGTTTGATAGC
L234F-R	GAACACGATCACAACCAAGAAAATGAGTTTGATAGC
L234W-R	GAACACGATCACAACCAACCAAATGAGTTTGATAGC
W154A-R	CACGAGAATAGCCGCTGCCGCAACTCCCAGAGAGATAGT
M138A-R	GGTTCTGTTGTTCGCGCTGTTGGCAGCCAGAAC
Y293A-R	TGCGAAGGCCGCGATCAGTGGGTTCAAACAGCA
V52A-R	AGCGAAGACCGCGAGCAGGTTGCCGACCAAACC
L70A-R	GAGGTTCAGCGCGTAGATGTCCGTAACACTCTT
F300A-R	GTGGTAGAGGTAACGGCGCGCCTTTTCACCTGCGAAGGC
N223A-R	CGCTTTCTTGTGCGCCTTACAGGAGAAGAGCGT
K299A-R	GTAGAGGTAACGGCGGAAAGCTTCACCTGCGAAGGC
D67A-R	CAGCAAGTAGATCGCCGTAACACTCTTAGGTTT
CCR5-WT DNA sequence	
GATTATCAAGTGTCAAGTCCAATCTATGACATCAATTATTATACATCGGAGCCCTGCCAAAAAATCAATGTGAAGC<br>AAATCGCAGCCCGCCTCCTGCCTCCGCTCTACTCACTGGTGTTCATCTTTGGTTTTGTGGGCAACATGCTGGTCAT<br>CCTCATCCTGATAAACTGCAAAAGGCTGAAGAGCATGACTGACATCTACCTGCTCAACCTGGCCATCTCTGACCT<br>GTTTTTCCTTCTTACTGTCCCCTTCTGGGCTCACTATGCTGCCGCCCAGTGGGACTTTGGAAATACAATGTGTCAA<br>CTCTTGACAGGGCTCTATTTTATAGGCTTCTTCTCTGGAATCTTCTTCATCATCCTCCTGACAATCGATAGGTACCT<br>GGCTGTCGTCCATGCTGTGTTTGCTTTAAAAGCCAGGACGGTCACCTTTGGGGTGGTGACAAGTGTGATCACTTG<br>GGTGGTGGCTGTGTTTGCGTCTCTCCCAGGAATCATCTTTACCAGATCTCAAAAAGAAGGTCTTCATTACACCTGC<br>AGCTCTCATTTTCCATACAGTCAGTATCAATTCTGGAAGAATTTCCAGACATTAAAGATAGTCATCTTGGGGCTGG<br>TCCTGCCGCTGCTTGTCATGGTCATCTGCTACTCGGGAATCCTtAAgACTCTGCTTCGGTGTCGAAATGAGAAGAA	

GAGGCACAGGGCTGTGAGGCTTATCTTCACCATCATGATTGTTTATTTTCTCTTCTGGGCTCCCTACAACATTGTCC<br>TTCTCCTGAACACCTTCCAGGAATTCTTTGGCCTGAATAATTGCAGTAGCTCTAACAGGTTGGACCAAGCTATGCA<br>GGTGACAGAGACTCTTGGGATGACGCACTGCTGCATCAACCCCATCATCTATGCCTTTGTCGGGGAGAAGTTCAG<br>AAACTACCTCTTAGTCTTCTTCCAAAAGCACATTGCCAAACGCTTCTGCAAATGCTGTTCTATTTTCCAGCAAGAG<br>GCTCCCGAGCGAGCAAGCTCAGTTTACACCCGATCCACTGGGGAGCAGGAAATATCTGTGGGCTTG	
WT-F	GACGATGATGACGATTATCAAGTGTCAAGTCCAATCTAT
T152A-R	CACAGCCACCACCCACGCGATCACACTTGTCACCAC
F238W-R	AACAATCATGATGGTCCAGATAAGCCTCACAGCCCT
DNGα_i-DNA sequence (S47C G202T G203A E245A A326S)	
ATGGGCTGCACGCTGAGCGCCGAGGACAAGGCGGCGGTGGAGCGGAGTAAGATGATCGACCGCAACCTCCGTG<br>AGGACGGCGAGAAGGCGGCGCGCGAGGTCAAGCTGCTGCTGCTCGGTGCTGGTGAATCTGGTAAATGTACAATT<br>GTGAAGCAGATGAAAATTATCCATGAAGCTGGTTATTCAGAAGAGGAGTGTAAACAATACAAAGCAGTGGTCTAC<br>AGTAACACCATCCAGTCAATTATTGCTATCATTAGGGCTATGGGGAGGTTGAAGATAGACTTTGGTGACTCAGCCC<br>GGGCGGATGATGCACGCCAACTCTTTGTGCTAGCTGGAGCTGCTGAAGAAGGCTTTATGACTGCAGAACTTGCTG<br>GAGTTATAAAGAGATTGTGGAAAGATAGTGGTGTACAAGCCTGTTTCAACAGATCCCGAGAGTACCAGCTTAATG<br>ATTCTGCAGCATACTATTTGAATGACTTGGACAGAATAGCTCAACCAAATTACATCCCGACTCAACAAGATGTTCT<br>CAGAACTAGAGTGAAAACTACAGGAATTGTTGAAACCCATTTTACTTTCAAAGATCTTCATTTTAAAATGTTTGAT<br>GTGACCGCCCAGAGATCTGAGCGGAAGAAGTGGATTCATTGCTTCGAAGGAGTGACGGCGATCATCTTCTGTGTA<br>GCACTGAGTGACTACGACCTGGTTCTAGCTGAAGATGAAGAAATGAACCGAATGCATGCCAGCATGAAATTGTTT<br>GACAGCATATGTAACAACAAGTGGTTTACAGATACATCCATTATACTTTTTCTAAACAAGAAGGATCTCTTTGAAG<br>AAAAAATCAAAAAGAGCCCTCTCACTATATGCTATCCAGAATATGCAGGATCAAACACATATGAAGAGGCAGCTG<br>CATATATTCAATGTCAGTTTGAAGACCTCAATAAAAGAAAGGACACAAAGGAAATATACACCCACTTCACATGTTC<br>CACAGATACTAAGAATGTGCAGTTTGTTTTTGATGCTGTAACAGATGTCATCATAAAAAATAATCTAAAAGATTGT<br>GGTCTCTTT	
DNGα_i-F	ATCGGGCGCGGATCCATGGGCTGCACGCTGAGCGCC
D350A-R	CTCGACAAGCTTTTAAAAGAGACCACAATCTTTTAG
